# The Use of Antioxidants for Cardiovascular Protection in Fetal Growth Restriction: A Systematic Review

**DOI:** 10.3390/antiox13111400

**Published:** 2024-11-15

**Authors:** Charmaine R. Rock, Suzanne L. Miller, Beth J. Allison

**Affiliations:** 1 The Ritchie Centre, Hudson Institute of Medical Research, 27-31 Wright Street, Clayton 3168, Australia; suzie.miller@monash.edu; 2 Department of Obstetrics and Gynaecology, Monash University, Clayton 3168, Australia

**Keywords:** fetal growth restriction, antioxidants, cardiovascular, brain sparing, developmental programming

## Abstract

Fetal growth restriction (FGR) increases the risk of cardiovascular disease. There are currently no treatment options available; however, antioxidants have shown potential to improve cardiovascular deficits associated with FGR. This systematic review aimed to determine whether antenatal antioxidant intervention can effectively protect the developing cardiovascular system in FGR. We searched for interventional studies that used an antenatal antioxidant intervention to improve cardiac and/or vascular outcomes in FGR published between 01/1946 and 09/2024 using MEDLINE and Embase (PROSPERO: CRD42024503756). The risk of bias was assessed with SYRCLE. The studies were assessed for cardiovascular protection based on the percentage of cardiac and/or vascular deficits that were restored with the antioxidant treatment. Studies were characterised as showing *strong cardiovascular protection* (≥50% restoration), *mild cardiovascular protection* (>0% but <50% restoration), an *antioxidant-only* effect (this did not include control group which showed a change with antioxidant intervention compared to FGR) or *no cardiovascular protection* (0% restoration). Thirty-eight publications met the inclusion criteria, encompassing 43 studies and investigating 15 antioxidant interventions. Moreover, 29/43 studies (71%) reported the restoration of at least one cardiac or vascular deficit with antioxidant intervention, and 21/43 studies (51%) were classified as strong cardiovascular protection. An ex vivo analysis of the arterial function in seven studies revealed endothelial dysfunction in growth-restricted offspring and antioxidant interventions restored the endothelial function in all cases. Additionally, four studies demonstrated that antioxidants reduced peroxynitrite-mediated oxidative stress. Notably, only 13/43 studies (32%) delayed antioxidant administration until after the induction of FGR. Antenatal antioxidant interventions show promise for providing cardiovascular protection in FGR. Melatonin was the most frequently studied intervention followed by nMitoQ, vitamin C and N-acetylcysteine, all of which demonstrated a strong capacity to reduce oxidative stress and improve nitric oxide bioavailability in the cardiovascular system of growth-restricted offspring; however, this systematic review highlights critical knowledge gaps and inconsistencies in preclinical research, which hinder our ability to determine which antioxidant treatments are currently suitable for clinical translation.

## 1. Introduction

Fetal growth restriction (FGR) describes the failure of a foetus to reach its biological growth potential. FGR occurs in 6–9% of pregnancies in high-income countries, but with a rate as high as 30% in low-resource countries [1]. Adverse maternal (malnutrition, pre-eclampsia), fetal (chromosomal abnormalities, multiple foetuses) or placental (poor remodelling of uteroplacental spiral arteries) conditions contribute to the development of FGR [2]. Despite their varying aetiology, a common causal factor is *placental insufficiency*, in which the transplacental delivery of oxygen and nutrients to the foetus is reduced and does not sustain normal fetal growth and development [3]. An inadequate oxygen supply creates a chronically hypoxic environment for the foetus which has known unfavourable impacts on the development of multiple organs and systems, including the heart and/or vasculature (hereafter combined to *cardiovascular*) [4,5], pulmonary [6] and neurological systems [7,8].

The link between a low birth weight and cardiovascular disease in adulthood was first described in the late 1980s as a phenomenon now known as the Developmental Origins of Health and Disease (DOHaD) hypothesis [4]. This work describes the association between an unfavourable intrauterine environment and adult-onset cardiovascular diseases such as coronary heart disease, ischemic heart disease and hypertension [9]. Whilst the mechanisms underpinning the developmental programming of cardiovascular disease are likely to be multifactorial and remain under investigation, oxidative stress is frequently found to play a mechanistic role [10,11,12,13,14].

Oxidative stress occurs when an overproduction of free radicals and/or reduced availability of endogenous antioxidants pushes the oxidative balance towards increased circulating or tissue concentrations of free radicals [15]. Even normal pregnancy is considered a state of elevated oxidative stress, due to the high metabolic rate and mitochondrial activity in the placenta producing excessive free radicals [16]. An increase in placental and fetal oxidative stress, beyond the normal levels seen in pregnancy, often occurs in response to placental dysfunction and subsequent fetal hypoxia [10,17]. It is hypothesised that oxidative stress plays a key role in the developmental programming of cardiovascular dysfunction. Dysregulated levels of free radicals can lead to direct cellular damage, as well as impair myocardial calcium handling [18], and reduce nitric oxide (NO) bioavailability in the vasculature [19,20,21], all of which contribute to the programming of cardiovascular dysfunction [11,22]. NO plays a significant role in the homeostatic regulation of blood pressure with its actions as a potent vasodilator; however, under hypoxic conditions, NO rapidly binds to the free radical, superoxide, to form the more potent free radical, peroxynitrite [23]. The speed of this reaction reduces the abundance of NO available to perform biological functions and exacerbates oxidative stress. Reduced NO bioavailability is widely acknowledged to be a fundamental factor in the onset and progression of many cardiovascular diseases [24], with preclinical [25,26,27] and clinical [22,28,29,30] studies demonstrating altered NO bioavailability in the context of FGR.

There is no cure or treatment for FGR; however, due to the pathogenic role of oxidative stress in the development of cardiovascular dysfunction in FGR, clinical and preclinical studies have explored whether restoring the oxidative balance with antioxidant supplementation in pregnancies affected by FGR could improve the cardiovascular function in the offspring. A wide range of preclinical animal models, including chronic hypoxia, NO deficiency, and maternal malnutrition, have been established to induce FGR. While each approach differs, they all replicate core pathophysiological processes and consequences central to FGR. Multiple pathological terms including FGR, intrauterine growth restriction (IUGR), placental insufficiency, hypoxia, and maternal malnutrition were, therefore, assessed. The objective of the current study was to systematically review the literature to determine the use and efficacy of antioxidants in the context of placental insufficiency and chronic fetal hypoxia to improve cardiovascular dysfunction in FGR foetuses and offspring. We searched for clinical and preclinical studies of *fetal growth restriction*, *placental insufficiency* and *fetal hypoxia* which utilised an *antenatal antioxidant* therapy to ameliorate *cardiovascular deficits*. We hypothesised that antioxidant supplementation would be associated with reduced oxidative stress and increased NO bioavailability, thus preventing cardiovascular deficits that are present in growth-restricted offspring.

## 2. Methods

This systematic review followed the guidelines of Preferred Reporting Items for Systematic Reviews and Meta-Analyses (PRISMA, Supplementary Tables S1 and S2) [31]. The review protocol was registered on PROSPERO (Registration number: CRD42024503756).

### 2.1. Search Strategy

We searched Medline (1946 to September 2024) and Embase (1947 to September 2024) via Ovid using the following strategy: (growth restriction OR growth retardation OR FGR OR IUGR OR placental insufficiency OR fetal hypoxia) AND (antioxidant). Searches were limited to English language articles.

### 2.2. Selection Criteria

Search results from Ovid were exported into Covidence Systematic Review Software (Veritas Health Innovation, Melbourne, Australia, available at http://www.covidence.org). Deduplication was conducted automatically using Covidence and manually by the study authors. Studies were required to meet the following selection criteria for inclusion: (1) randomised clinical trial or preclinical model investigating chronic fetal hypoxia or placental insufficiency; (2) intervention program was antenatal antioxidant treatment; (3) fetal or postnatal cardiovascular outcomes were assessed. Studies conducted in vitro (cell or tissue culture) or those focused solely on the placental vasculature were excluded from further review. Using Covidence, titles and abstracts of retrieved studies were screened independently by two study authors (CRR and BJA) to identify studies that met the inclusion criteria. Any publications that did not meet the inclusion criteria were excluded from further review. Any disagreements were resolved through discussion with an additional reviewer (SLM). Full texts of potentially eligible studies were then retrieved and independently assessed for eligibility by two authors (CRR and BJA) based on the inclusion criteria, with any disagreements resolved by a third reviewer (SLM).

### 2.3. Data Extraction

Data were extracted from included studies by two authors (CRR and BJA) independently via Covidence. Extracted information included author and publication year, animal characteristics including species and strain, model of FGR, gestational age when FGR was induced and control group parameters. Details of each intervention were extracted including the dose, timing, route and frequency of administration and vehicle. Data on outcome measures were exacted from each study, including cardiac or vascular morphology, in vivo cardiovascular function, ex vivo cardiac and/or vascular function and molecular analyses of antioxidant mechanisms. The gestational or postnatal age for outcome measures was recorded. Principally, studies were categorised by level of cardiovascular protection. Any studies that showed no improvement of cardiovascular deficits with antioxidant treatment were categorised as ‘no cardiovascular protection’. Studies that demonstrated restoration of cardiovascular deficits to a degree > 0% but <50% with antioxidants were labelled ‘mild cardiovascular protection’, whereas studies that reduced cardiovascular deficits ≥ 50% with antioxidant treatment were termed ‘strong cardiovascular protection’. Any studies that did not include a non-FGR control group or the FGR group did not demonstrate any cardiovascular deficits compared to the control group were assessed for an antioxidant effect. Studies were deemed to have an ‘antioxidant only’ effect if the antioxidant treatment altered one of the assessed cardiovascular outcomes compared to the FGR group.

### 2.4. Risk of Bias

Two study authors (CRR and BJA) independently assessed the risk of bias for each included study using the Systematic Review Centre for Laboratory Animal Experimentation (SYRCLE) risk of bias tool for animal studies [32] and the Cochrane risk of bias tool for randomised trials for human studies. SYRCLE assesses whether a study is free from selection bias, performance bias, detection bias, attribution bias and reporting bias, reported as “Yes, No or Unclear”. Disagreements were resolved through discussion with an additional author (SLM).

## 3. Results

**Figure 1:**
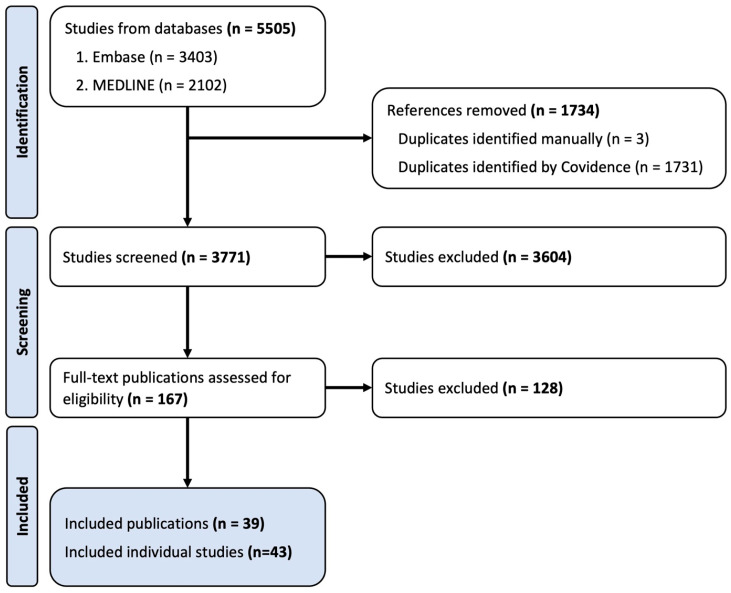
Flow diagram depicting number of studies in each stage of the selection process.

In total, 5505 records were identified through the described search procedure. Following the removal of duplicates, 3771 publications were screened by the title and abstract where 3604 records were excluded from further review. Full-text screening for eligibility was performed on 167 records and, based on the inclusion and exclusion criteria, 38 studies were included in this systematic review. Three of the included publications were subdivided into two studies as they included multiple study designs (e.g., two different models of FGR) within the one publication. The final number of studies examined in this systematic review is 43 (Figure 1) [26,27,33,34,35,36,37,38,39,40,41,42,43,44,45,46,47,48,49,50,51,52,53,54,55,56,57,58,59,60,61,62,63,64,65,66,67,68,69]. For brevity, studies will be discussed by outcome measures in the results section, with full details provided in Table 1 and Table 2.

### 3.1. Characteristics of Included Studies

The characteristics of the included studies are outlined in Table 1. Small animals were most frequently used (rats, n = 15 [34,35,39,42,43,44,46,48,49,54,55,59,63,64,68]; mice, n = 5 [45,66,67]; guinea pigs, n = 5 [33,50,53,60,62]), comprising 58% (25/43) of the included studies. The remaining studies were conducted in sheep (28%, n = 11) [26,37,38,40,41,47,56,57,61,65], pigs (7%, n = 3) [51,52,69] and developing chicken embryos (7%, n = 3) [27,37,58]. One human randomised clinical trial was eligible for inclusion based on the selection criteria [36].

Of the preclinical studies included, 12 separate experimental techniques were used to induce FGR. Hypoxic chambers were used to house pregnant animals [33,34,35,37,38,39,42,43,44,46,48,49,50,54,55,59,68] or fertilised chicken eggs [27,37,58] during gestation in 20 studies (47%). Two studies (5%) used a high-altitude pregnancy model in sheep [40,47] to induce FGR via maternal hypoxemia and subsequent fetal hypoxemia. Maternal malnutrition was induced in four studies (9%) by restricting the maternal food intake to 50–60% of their daily nutrient requirement [51,52,61,69], whilst one study (2%) allowed pregnant ewes to be housed and graze on a Chilean–Patagonian prairie that does not adequately provide the nutritional requirements of the pregnant sheep [65]. The placental-specific insulin-like growth factor 2 knockout (P0^+/−^) mouse model was used in one study (2%) to induce placental dysfunction and pathology [67]. The endothelial NO synthase knockout (eNOS^−/−^, 7%, n = 3) [45,66,67] and Catechol-O-methyl transferase knockout (COMT^−/−^, 2%, n = 1) [66] mouse models were used to create hypertensive dams, mimicking clinical pre-eclampsia accompanied by placental insufficiency. Seven studies (16%) induced placental insufficiency via single umbilical artery ligation (12%, n = 5) [26,41,56,57] or progressive uterine artery occlusion (5%, n = 2) [53,60] to reduce the placental function and blood flow to the foetus leading to a reduction in both oxygen and the nutrient delivery to the foetus. Complicated and poorly controlled diabetes in pregnant women can also lead to FGR and, therefore, one study used the Cohen diabetic rat model to induce FGR by feeding pregnant Cohen diabetes-sensitive rats a high-sucrose, low-copper diet [64]. The daily administration of the anticonvulsant, phenytoin, to pregnant rats was used in one study (2%) to induce chronic intrauterine hypoxia [63]. Finally, one study (2%) in guinea pigs used a spontaneous FGR model where guinea pigs were classified as FGR if they had a body weight of <85 g and a brain-to-liver ratio of >0.65 at the time of tissue collection [62]. The human clinical trial used the Delphi consensus definition of early-onset FGR [70] for inclusion in the study [36].

Based on the Delphi consensus definition, FGR is classed as early-onset when diagnosed before 32 weeks of gestation (0.78 of gestation). All included studies induced FGR before 0.78 of gestation; however, four studies (9%) surgically induced FGR in fetal sheep at 0.71 of gestation [26,41] and this was categorised as late-onset FGR by the authors [71]. Additionally, Renshall et al. [67] stated that the placental-specific insulin-like growth factor 2 knockout model is a model of late-onset FGR as this model does not present with abnormalities in the uteroplacental blood flow, but demonstrates a placental pathology consistent with the clinical presentation of late-onset FGR.

There was an even distribution of studies that investigated fetal cardiovascular outcomes (46%, n = 20) [26,27,33,36,37,45,50,52,53,56,57,58,61,62,64,65,66,67] compared to postnatal cardiovascular outcomes (41%, n = 17) [26,34,35,39,40,41,42,43,44,48,51,54,55,59,63,69] and six studies (14%) [37,38,46,49,60,68] reported cardiovascular outcomes in both fetal and postnatal animals. Fourteen (33%) studies that included postnatal time points conducted cardiovascular assessments in adulthood. Eight studies (19%) did not include a control group, which reduced our ability to determine the FGR-induced impact on the cardiovascular system and we could not determine whether antioxidant treatment normalised the outcomes [36,40,47,51,52,54,65,69]. For the classification of potential cardiovascular benefits of interventions in Table 2, these studies were assessed based on whether an antioxidant effect was demonstrated.

### 3.2. Therapeutic Interventions, Doses and Regimens

For the 43 studies, 15 antioxidant interventions were investigated. The most frequently used antioxidant interventions were melatonin (n = 11, 26%) [26,40,41,47,49,58,61,63,67], MitoQ-loaded nanoparticles (nMitoQ, n = 5, 12%) [34,44,48,54,55] and vitamin C (n = 4, 9%) [38,39,46,59] and N-acetylcysteine (n= 4, 9%) [33,50,53,60]. Three studies (7%) used sildenafil [27,56,57] and three studies used MitoQ (7%) [37,68]. Three studies (7%) used a combined therapy of two antioxidants (linseed oil and hydroxytyrosol (n = 2, 5%)) [51,52] and vitamins C and E (n = 2, 5%) [65] and two studies (5%) used the polyamine spermidine [42,43]. Antenatal resveratrol supplementation was used in two different mice models of placental insufficiency [66]. All other interventions (hydroxytyrosol alone [69], allopurinol [35], pyrroloquinoline quinone [62], pentoxifylline [36] and pomegranate juice [45]) were investigated in one of the included studies.

Most studies gave continuous or daily repeated doses of the intervention (88%, n = 38) until the termination of pregnancy (fetal studies) [26,27,33,36,37,45,50,52,53,56,57,58,60,61,62,64,65,66,67] or birth (postnatal studies) [26,35,37,38,39,40,41,42,43,46,47,49,51,59,60,63,68,69]. All five studies that investigated nMitoQ (12%) gave a single 125µM bolus injection via the rat tail vein on the same day that FGR was induced [34,44,48,54,55]. In 20 studies (47%), the treatment regimen began on the same day that FGR was induced [26,35,37,38,39,41,42,43,46,49,51,52,59,61,64,66,68,69], whereas six studies (14%) in sheep and guinea pigs delayed the administration of the intervention from two to five days following the induction of FGR [26,33,50,56,57,65]. Three studies (7%) were conducted in rodents [45,67], three studies (7%) in fertilised chicken eggs [27,37,58] and two studies (5%) in pregnant sheep at a high altitude [40,72], where the induction of FGR began at the start of gestation and the administration of the intervention began at approximately 0.63 of gestation through to birth or the gestational endpoint [37,40,45,47]. Three studies (7%) commenced the intervention before the induction of FGR, including two in which guinea pigs were treated with N-acetylcysteine the day prior [53,60] and another giving melatonin from the beginning of gestation [63]. Finally, the human clinical trial began pentoxifylline treatment at a diagnosis of FGR until delivery [36].

The most predominant route of administration was oral, either through drinking water or food supplementation (60%, n = 26) [33,35,36,39,40,45,46,47,49,50,51,52,53,59,60,61,62,63,64,65,66,67,68,69], and in all other studies, an intravenous (35%, n = 15) [26,27,34,37,38,41,42,44,48,54,55,56,57,58] or intraperitoneal (5%, n = 2) [42,43] injection of the intervention was used.

### 3.3. Overall Potential for Cardiovascular Protection

**Figure 2:**
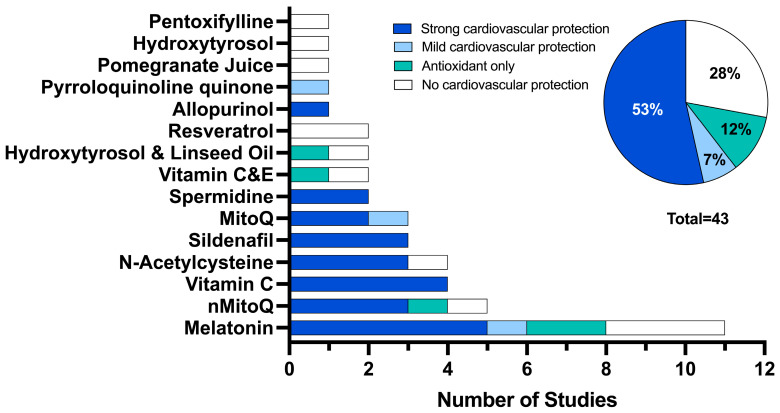
The number of studies that demonstrated cardiovascular protection: categorised as demonstrating strong cardiovascular protection (≥50% of deficits improved with antioxidant intervention, dark blue), mild cardiovascular protection (>0% but <50% of deficits improved with antioxidant intervention, light blue), antioxidant effect only (studies that did not include a non-FGR control group which showed a change with antioxidant intervention compared to FGR, teal) and no cardiovascular protection (0% of outcomes improved with antioxidant intervention, white).

Each study was evaluated for the level of cardiovascular protection via reduction of cardiovascular deficits or via an antioxidant effect on the assessed cardiovascular outcomes (Figure 2). Twenty-three studies (53%) demonstrated a strong cardiovascular protection effect using melatonin (12%, n = 5) [26,41,49,58], vitamin C (9%, n = 4) [38,39,46,59], nMitoQ (7%, n = 3) [34,48,55], N-acetylcysteine (7%, n = 3) [33,53,60], sildenafil (7%, n = 3) [27,56,57], MitoQ (5%, n = 2) [37], spermidine (5%, n = 2) [42,43] and allopurinol (2%, n = 1) [35]. Only three studies (7%) showed a mild cardiovascular protection effect using melatonin (2%, n = 1) [61], MitoQ (2%, n = 1) [68] and pyrroloquinoline quinone (2%, n = 1) [62]. Five studies demonstrated an antioxidant effect only using melatonin (5%, n = 2) [40,67], nMitoQ (2%, n = 1) [54], vitamin C and E (2%, n = 1) [65] and hydroxytyrosol and linseed oil (2%, n = 1). Finally, no cardiovascular protection was observed in 12 studies (28%) using melatonin (7%, n = 3) [47,63,67], resveratrol (5%, n = 2) [66], nMitoQ (2%, n = 1) [44], N-acetylcysteine (2%, n = 1) [50], vitamin C and E (2%, n = 1) [64], hydroxytyrosol and linseed oil (2%, n = 1) [51], hydroxytyrosol (2%, n = 1) [69], pomegranate juice (2%, n = 1) [45] and pentoxifylline (2%, n = 1) [36].

### 3.4. Cardiovascular Function

#### 3.4.1. Umbilical Artery Pulsatility Index

A raised umbilical artery pulsatility index occurs when there is a decreased end-diastolic flow due to reduced placental perfusion and is considered a marker of fetal compromise that has formed part of the consensus definition of FGR since 2016 [70]. Asadi et al. [36] conducted a randomised clinical trial assessing pentoxifylline for severe early-onset FGR with an ultrasound to determine the fetal umbilical artery and middle cerebral artery pulsatility index prior to delivery. This study did not include a non-FGR control cohort; however, the diagnosis of FGR was determined by the Delphi consensus which includes an umbilical artery pulsatility index > 95th percentile as one of the parameters of FGR. Pentoxifylline administration from 28–31 weeks of gestation did not alter the umbilical artery or middle cerebral artery pulsatility index. Herrera et al. [53] showed that FGR was associated with a higher umbilical artery pulsatility index in guinea pigs, which progressively improved during gestation in response to N-acetylcysteine treatment. Two studies (5%) in knockout mice showed that the blood flow through the umbilical artery was not altered with FGR and resveratrol treatment did not result in any additional effects [66].

#### 3.4.2. Cardiac Function

Cardiovascular outcomes from all studies are outlined in Table 2. Heart rate was the most common functional cardiovascular outcome with nine studies reporting the heart rate in vivo (16%, n = 7) [34,38,40,57,59,61,68] or ex vivo (5%, n = 2) [26,46]. However, FGR did not alter the heart rate in eight of the nine studies. Lemley et al. [61] showed a decrease in the heart rate with FGR, while melatonin did not restore the heart rate in this study. Seven studies investigated the cardiac function ex vivo using Langendorff techniques [26,37,46,54,55,58,68], for which five studies looked at the left ventricular developed pressure [26,37,46,58,68] and four assessed the maximum rate of a cardiac contraction [26,46,58,68]. Three of these studies did not show any change in the left ventricular developed pressure with FGR, whilst two showed a reduction in the left ventricular developed pressure with FGR, which was restored with melatonin [58] and MitoQ [37]. Similarly, three of the studies did not demonstrate any change in the maximum rate of contraction [26,58,68], but one study showed that FGR increased the maximum rate of contraction, which was ameliorated by antenatal vitamin C [46]. Conversely, Hula et al. [54,55] used the Langendorff system to assess the ability of the isolated heart to recover from ischaemia and showed in one study that nMitoQ treatment improved baseline cardiac recovery following 20 min of global ischaemia [55].

#### 3.4.3. Blood Pressure

Inocencio et al. [57] showed an increase in the fetal blood pressure following the induction of placental insufficiency in fetal sheep and this was restored with sildenafil treatment [57]. Five studies (12%) investigated the mean arterial blood pressure in adult animals who were the subject of a hypoxic pregnancy [34,37,38,59,68]. Three (7%) of these studies were conducted in rats and none of these studies indicated that a hypoxic pregnancy altered the mean blood pressure [34,59,68]. MitoQ [68] and nMitoQ [34] had no further impact on the blood pressure; however, vitamin C decreased the blood pressure [59]. The two remaining studies (5%), conducted in sheep, showed that adult offspring following a hypoxic pregnancy had an increased blood pressure compared to control sheep and this was ameliorated with vitamin C [38] and MitoQ [37]. Gonzalez-Candia et al. [47] investigated the pulmonary arterial pressure, vascular resistance and cardiac output in newborn lambs from postnatal day 3 to 12 and showed that antenatal melatonin did not alter either variable. The baroreceptor reflex, which is the homeostatic mechanism that maintains the blood pressure, was assessed by Kane et al. [59] in adult rats, with this study demonstrating that there was an increased baroreflex gain in rats of a hypoxic pregnancy and this was normalised with vitamin C. The carotid and femoral vascular resistance index was measured in adult growth-restricted guinea pigs, which showed no differences in the carotid artery, but vascular resistance was significantly increased in the femoral artery which was normalised with antenatal N-acetylcysteine.

#### 3.4.4. Endothelium-Independent Vascular Dilation

The aorta (2%, n = 1) [53], umbilical (9%, n = 4) [45,53,67], femoral (14%, n = 6) [27,37,46,56,58,60], carotid (2%, n = 1) [60], middle cerebral (5%, n = 2) [40,56] and coronary (2%, n = 1) [26] arteries were isolated for an ex vivo myography to assess the endothelium-independent vasodilatory capacity to the NO donor, sodium nitroprusside (SNP), in 11 (26%) studies. Of these studies, two were conducted in knockout mice (eNOS^−/−^ and P0^+/−^) and showed no changes in the umbilical reactivity to sodium nitroprusside with FGR; however, the overall vasodilation was increased by melatonin [67] and pomegranate juice [45] supplementation. In contrast, Renshall et al. [67] showed that umbilical arteries from eNOS^−/−^ mice displayed increased reactivity to SNP and this was not restored with melatonin. Herrera et al. [53] demonstrated a similar phenotype whereby umbilical arteries from growth-restricted guinea pigs were more sensitive to SNP, and this was improved with N-acetylcysteine treatment. In the same study, the aorta from growth-restricted fetal guinea pigs showed no difference in reactivity to SNP compared to the controls; however, treatment with N-acetylcysteine reduced the overall endothelium-independent vasodilation [53]. Coronary arteries isolated from newborn growth-restricted lambs showed no difference in reactivity to SNP and no further change was observed with melatonin [26]. The vasodilatory response to SNP in middle cerebral arteries was increased in growth-restricted sheep foetuses; however, sildenafil treatment increased vasodilation to SNP [56]. Additionally, antenatal melatonin increased the sensitivity to SNP in middle cerebral arteries of neonatal growth-restricted lambs compared to untreated FGR lambs [40]. Krause et al. [60] assessed carotid vasodilation to SNP in fetal and adult guinea pigs and found no difference in fetal FGR guinea pigs; however, by 8 months old, SNP-mediated vasodilation was reduced and this was improved with antenatal N-acetylcysteine. Three studies (7%) [37,46,60] showed impaired endothelium-independent vasodilation in the femoral artery and this was restored with MitoQ [37], but not vitamin C [46] or N-acetylcysteine [60]. Vascular responses to SNP in femoral arteries of hypoxic chicken embryos were not altered by FGR, melatonin [58] or sildenafil [27]. Femoral arteries isolated from growth-restricted fetal sheep exhibited an increased responsiveness to SNP compared to the controls [56]. However, treatment with sildenafil significantly reduced this responsiveness, resulting in impaired vasodilation with SNP in sildenafil-treated foetuses compared to the control lambs [56].

#### 3.4.5. Endothelium-Dependent Vascular Dilatation

Endothelial dysfunction is an early predictor of cardiovascular disease [73] and nine studies (21%) assessed the endothelial function in isolated femoral (19%, n = 8) [27,35,37,38,46,56,58,60] and mesenteric (2%, n = 1) [34] arteries using ex vivo wire myography. Eight of the nine studies demonstrated endothelial dysfunction; however, Aljunaidy et al. [34] only showed endothelial dysfunction in mesenteric arteries from 13-month-old hypoxic females, but not in male hypoxic offspring or 7-month-old animals of either sex. All seven studies indicated that antioxidant treatment (melatonin [58], vitamin C [46], N-acetylcysteine [60], sildenafil [27], MitoQ [34,37], allopurinol [35]) restored the endothelial function to the control levels. Inocencio et al. [56] showed that FGR did not alter the endothelial function in the femoral artery at 0.85 of gestation; however, treatment with antenatal sildenafil significantly impaired the endothelial function. Endothelium-dependent vasodilation was impaired in isolated aorta from fetal guinea pigs [53] and coronary arteries from newborn lambs [26] and improvements were observed with N-acetylcysteine and melatonin, respectively. Isolated middle cerebral arteries from 12-day-old lambs treated with antenatal melatonin [40] displayed increased endothelium-dependent vasodilator sensitivity compared to untreated lambs of a hypoxic pregnancy. Growth-restricted guinea pigs displayed a normal carotid endothelial function at 0.91 of gestation; however, by 8 months old, endothelial dysfunction had developed, and antenatal N-acetylcysteine restored the endothelial function to the control levels. Tare et el. [26] used pressure myography to show that endothelium-derived NO bioavailability was decreased in newborn growth-restricted lambs, but with the restoration of NO following antenatal melatonin treatment.

#### 3.4.6. Vascular Contraction

Phenylephrine was used in six studies (14%) to assess the contractility of peripheral vascular beds [27,34,38,49,58,68]. Of these studies, in vivo techniques were used in two studies (5%) [38,68] and ex vivo wire myography in four studies (9%) [27,34,49,58]. The in vivo assessment showed that the femoral artery is more sensitive to phenylephrine in offspring exposed to hypoxia during gestation, and this was restored with vitamin C [38] and MitoQ [68]. Similarly, mesenteric arteries isolated from adult rats of a hypoxic pregnancy were more reactive to phenylephrine and this was improved with continuous oral maternal melatonin [49], but was exacerbated by one antenatal injection of nMitoQ [34]. Two studies showed that exposure to hypoxia in utero did not alter the vasoreactivity to phenylephrine in isolated femoral arteries from chicken embryos and with no further alteration with sildenafil or melatonin [27,58]. In one study, pressure myography was used to show that stiffness in the coronary artery wall was increased with FGR, but this was ameliorated with melatonin [26].

### 3.5. Cardiovascular Morphology

Heart weight or heart:body weight were the most frequent morphological outcomes reported (n = 20, 47%). Of these, 11 studies (26%) found no change to the heart weight in FGR offspring and with no further change with antenatal antioxidants [26,38,46,49,51,52,55,61,62,64,69]. Two studies (5%) showed a decreased heart weight and one (2%) study showed an increase in the relative heart weight with FGR [27,33,58]. The heart weight was not normalised by antenatal antioxidants (sildenafil, melatonin and N-acetylcysteine) [27,33,58]. Three studies (7%) also indicated a decrease in the heart weight with FGR; however, antioxidant treatment (N-acetylcysteine [53] and spermidine [42,43]) restored the heart weight. Similarly, one study (2%) demonstrated a decrease in the heart weight which was normalised with N-acetylcysteine [50]. In two studies (5%), antioxidants (hydroxytyrosol and linseed oil [52], and vitamins C and E [65]) decreased the heart weight relative to FGR, but there was no control group to determine if this was a normalisation to control levels.

The left ventricular volume or area was assessed in three (7%) studies. Itani et al. [58] demonstrated that hypoxic chicken embryos had an increased left ventricular volume that was not improved with melatonin. Two additional studies showed that neither FGR or antenatal antioxidants altered the fetal left ventricular area [46,49]. Hansell et al. [49] showed that the left ventricular wall:lumen area was decreased in 4-month-old rats of a hypoxic pregnancy and this was restored with melatonin. The fetal aortic wall:lumen area was increased in adult rats of a hypoxic pregnancy in two studies and normalised with vitamin C [46] and melatonin [49]. Conversely, the fetal aortic wall:lumen area was decreased in hypoxic chicken embryos and this reduction persisted with antenatal melatonin treatment.

Three studies (7%) examined the integrity of the cerebral vasculature, showing decreased blood vessel abundance [39,41] and a compromised blood–brain barrier structure [41]. Camm et al. [39] demonstrated that this reduced the vascular density in the hippocampus of 4-month-old FGR rats treated with vitamin C. Contrastingly, Castillo-Melendez and colleagues [41] did not see an improvement in the number of blood vessels in the white matter in one-day-old FGR lambs treated with melatonin; however, melatonin significantly improved the integrity of the blood–brain barrier, with improved pericyte and astrocyte endfeet coverage, as well as reduced microbleeds compared to untreated FGR lambs. Candia et al. [40] assessed the diameter and area of the middle cerebral artery from neonatal FGR lambs treated with antenatal melatonin and found that melatonin did not impact the middle cerebral artery morphology; however, it is uncertain if FGR itself caused a change in the middle cerebral artery structure as no control group was included.

### 3.6. Protein and Molecular Analyses in Cardiovascular Tissue

Fourteen studies (34%) conducted protein and/or molecular analyses on cardiovascular tissue, with most studies focused on markers of oxidative stress and antioxidant activity. The most frequently investigated biomarker of oxidative stress was 3-nitrotyrosine (n = 5, 12%) [27,40,46,53,58], which was increased in the FGR cohorts from four of these studies. Two of the studies indicated that N-acetylcysteine [53] and vitamin C [46] reduced 3-nitrotyrosine in the fetal and adult aorta, respectively, whereas one study showed that melatonin reduced 3-nitrotyrosine in the chicken embryo heart [58]. One study showed that melatonin had no impact on 3-nitrotyrosine in the middle cerebral artery of newborn lambs [40] and another showed no changes in the chicken embryo heart with sildenafil [27]. Another marker of oxidative stress, 4-hydroxynonenal (4-HNE), was investigated in cardiac tissue in three studies [27,49,58], wherein two studies demonstrated an increase in 4-HNE in fetal hearts of hypoxic chicken embryos that was restored with antioxidants (melatonin [58] and sildenafil [27]). Hansell et al. [49] assessed 4-HNE expression in fetal and 4-month-old hearts and saw no changes with FGR or melatonin. Al-Hasan et al. [33] assessed oxidative stress levels using malondialdehyde, which were increased in the cardiac tissue from hypoxic fetal guinea pigs and restored with N-acetylcysteine treatment. The levels of antioxidant enzymes such as superoxide dismutase, catalase and glutathione peroxidase were measured in cardiac tissue in five studies (12%) [27,42,43,58,68]. FGR decreased superoxide dismutase expression in the heart in four studies [27,42,43,58] and remained unchanged in one study [68]; however, only spermidine restored superoxide dismutase expression [42,43], with melatonin [58], sildenafil [27] and MitoQ [68] having no impact on superoxide dismutase levels. Catalase expression in hearts collected from chicken embryos was reduced in two studies, with antioxidant (melatonin [58] and sildenafil [27]) supplementation having no impact. However, one study in rats showed no differences in the catalase in the growth-restricted fetal heart, but by 4 months of age, there was increased catalase expression in the heart which was improved with antenatal MitoQ treatment [68]. In hearts from 1-day-old hypoxic rats, catalase expression was decreased and this was restored with spermidine [43]. The glutathione peroxidase levels were unchanged in fetal cardiac tissue from hypoxic chickens; however, they were increased with the treatment of melatonin [58] and sildenafil [27]. Spiroski et al. [68] showed no changes in glutathione peroxidase levels in the heart of fetal rats; however, there was an increase in glutathione peroxidase at 4 weeks of age which was not ameliorated with MitoQ. NO bioavailability was assessed with molecular techniques in two studies in fertilised chicken eggs by determining the fetal cardiac nitrate and nitrite concentration [27,58]. Both studies showed an increase in the nitrate and nitrite concentration in FGR embryos, with both melatonin and sildenafil returning concentrations to control levels [27,58].

## 4. Discussion

It has long been established that being growth-restricted in utero increases the risk of developing cardiovascular disease in adulthood [4,9], with clinical and preclinical research in the past two decades demonstrating that subclinical signs of cardiovascular dysfunction are detectable in childhood [74,75,76] and even in the neonatal period [5,19,77,78]. However, there are currently no treatment options available for FGR. Pre-clinical research has determined a key role for oxidative stress as a contributing factor in the programming of cardiovascular disease [26,38,46,79]. Despite decades of interest in the use of antioxidants to combat oxidative stress in FGR, it remains unclear whether antenatal antioxidants are truly protective for the cardiovascular system, if they protect the mechanisms through which they exert this protection and which specific antioxidant treatments offer the most benefit. Therefore, this current analysis aimed to systematically review the literature to determine whether antenatal antioxidant supplementation is protective for the cardiovascular system of growth-restricted offspring and to identify knowledge gaps in the literature that require further investigation. A total of 43 studies were identified, with 15 different antioxidant treatments evaluated, for which 11 showed varying degrees of protective potential for the developing cardiovascular system.

### 4.1. Antioxidant Treatment Potential

Overall, two-thirds of the identified studies showed cardiovascular protection or an antioxidant effect with an antenatal antioxidant intervention. Of these, almost half of all included studies showed that antenatal antioxidant treatment showed strong cardiovascular protection on the outcomes assessed. Most strikingly, antioxidants proved effective at combating endothelial dysfunction, which is a well characterised early predictor of cardiovascular disease and is one of most established vascular complications associated with FGR [22,27,34,35,38,46,58,80]. All studies that assessed the endothelial function in isolated peripheral arteries demonstrated endothelial dysfunction associated with FGR, and showed that antenatal treatment with melatonin, vitamin C, N-acetylcysteine, sildenafil, MitoQ and allopurinol normalised the endothelial function. The same deficits in blood vessel function were not seen when other vessels were examined, for example, in the aorta [53], middle cerebral artery [56] and the umbilical artery [45,67]. The region-specific responses to chronic hypoxia with brain sparing likely reflect the differential vascular control of essential and non-essential vascular beds and their roles during periods of chronic hypoxia. During chronic brain sparing, peripheral (non-essential) arteries are vasoconstricted, while arteries supplying essential organs such as the umbilical cord and aorta do not experience vasoconstriction [81,82]. Thus, brain sparing alters the developmental environment for each vascular bed with non-essential fetal beds developing whilst chronically vasoconstricted, and non-essential vascular beds developing exposed to an increased shear due to an increased preload.

A reduction in the local NO concentration has a significant impact on the local blood vessel tone, wherein reduced local NO leads to increased vasoconstriction, which in turn can impact the blood pressure. It is due to this vital role that reduced NO bioavailability is considered the leading factor contributing to the development of endothelial dysfunction [23,30]. There is significant evidence that peripheral arteries have reduced NO bioavailability [27,38,58]. Reduced NO bioavailability has long been hypothesised to be a result of exposure to low levels of oxygen (exacerbated by the reduced blood flow in this vascular bed) and high levels of the free radical, superoxide [83]. Superoxide dismutase is the key regulator of superoxide levels and is decreased in cardiovascular tissue from FGR animals [27,42,43,58]; however, only spermidine demonstrated an ability to normalise cardiac superoxide dismutase. NO can compete with superoxide dismutase for superoxide interactions to form the more potent free radical peroxynitrite [20]. 3-nitrotyrosine is a downstream product of peroxynitrite-mediated oxidative stress, which was found in these studies to be upregulated in cardiovascular tissue following FGR. This review highlights that antioxidant interventions can diminish peroxynitrite-induced damage [46,53,58], but this is not mediated by an improvement in superoxide dismutase levels. This may indicate that antioxidant interventions have a more direct scavenging effect on superoxide, rather than upregulating antioxidant enzymes. Jackson et al. [84] previously demonstrated that although vitamin C can scavenge superoxide at low concentrations, endothelium-mediated vasodilation only resulted from high concentrations of vitamin C. Although there is great variance between interventions and regimes, many of the studies demonstrated the ability of antioxidants to improve cardiovascular deficits when high doses of the intervention were applied [26,41,49,53,57,60]. This is important because, despite being naturally occurring compounds, antioxidants may not be entirely safe at high concentrations. A recent systematic review of melatonin treatment in adults showed that high doses of melatonin (≥10 mg) did not increase the risk of severe adverse events. However, it did increase the risk of adverse events such as drowsiness or headaches [85]. In the case of pregnancy complications, both maternal and fetal health must be carefully considered, but overall, this review confirms the effectiveness of antenatal antioxidants for counteracting the programming of endothelial dysfunction in FGR by improving NO bioavailability.

Melatonin, vitamin C, nMitoQ and N-acetylcysteine were the most frequently used antioxidants, and they also demonstrated the most promising ability to protect against cardiovascular deficits. Melatonin, vitamin C, nMitoQ and N-acetylcysteine all have strong antioxidant properties; however, they also exhibit anti-inflammatory properties, and this is critical given that inflammation is likely to play a role in the pathogenesis of cardiovascular disease in FGR [86]. Oxidative stress and proinflammatory processes are closely linked as the overproduction of free radicals induces an inflammatory response and many immune cells produce free radicals once activated [87]. Although these frequently studied antioxidant treatments defend against both oxidative stress and inflammatory processes, none of them demonstrated the ability to protect against all cardiovascular deficits that were assessed across the studies. This is likely due to the complexity and multifactorial nature of FGR.

### 4.2. Inducing FGR

#### 4.2.1. Heterogeneity in FGR

The primary causes of FGR are diverse and multifactorial, arising from placental, maternal, fetal and/or environmental factors, but a common pathology for these is placental insufficiency. Clinically, many cases of FGR have two or more complications, for example, pre-eclampsia and gestational hypertension. There is also a spectrum of the severity of the consequences associated with FGR, influenced by the timing of onset of placental insufficiency (early- or late-onset), the severity of placental dysfunction and the gestational age at birth [88]. The majority of studies assessed in this systematic review induced early-onset FGR (diagnosis < 32 weeks’ gestation), likely due to its association with more severe outcomes, including a high incidence of prematurity. Preclinical animal models cannot produce the complexity of clinical FGR, but the diversity of the animal models used allows researchers to characterise fundamental pathways and mechanisms that contribute to altered growth and cardiovascular development, and to test interventions such as antioxidants across different animal models.

In this systematic review, more than half of the studies directly caused hypoxia during pregnancy, about 25% induced placental insufficiency and 14% of studies used a model of maternal malnutrition. We consider that the use of multiple animal models to cause FGR via different means is a strength of preclinical research. Placental insufficiency is said to be the primary cause of FGR [89] and suboptimal placental function is a diagnostic criteria for FGR [70]. In preclinical studies, Herrera and colleagues induced placental insufficiency, as evidenced by the high umbilical artery pulsatility index by progressively occluding the uterine artery in pregnant guinea pigs [53]. Causing placental insufficiency in preclinical studies provides clinical relevance to determine how fetal hypoxemia, hypoglycaemia and the brain sparing response act to alter cardiovascular development. In a further study in the same model, Krause and colleagues [60] demonstrated increased femoral vascular resistance in vivo and ex vivo, and femoral endothelial dysfunction was present in adult offspring of growth-restricted pregnancies. Additionally, the progressive uterine artery occlusion model in guinea pigs successfully mimics many previously described cardiovascular outcomes in FGR [90]. In comparison to rodents, sheep and pigs, the guinea pig placenta most strongly resembles the human placenta as it is haemochorial and the process of trophoblast invasion is homologous to humans [90]. The form of placentation is important when considering maternal antioxidant supplementation and the ability of the antioxidant to either act on the placenta or cross the placenta to the foetus. Almost all the interventions assessed in this review have been shown to cross the placenta, with the exception of spermidine and pyrroloquinoline quinone. However, due to their small molecular size, both spermidine and pyrroloquinoline quinone may indeed cross the placenta. Furthermore, since FGR most commonly results from placental dysfunction, the intended target for some antioxidants is the placenta itself, with the aim of improving the placental function and oxygen and nutrient delivery to the foetus.

#### 4.2.2. Heterogeneity of Cardiovascular Outcomes

We also noted the heterogeneity of outcomes, with some studies specifically focused on the heart, while other studies were focused on vascular outcomes related to essential and/or non-essential vascular beds. The brain sparing response to fetal hypoxia redistributes the cardiac output to essential organs such as the brain, which in turn has differential vascular haemodynamic effects throughout the body. In this state, the fetal heart is working against an increased systemic afterload, the non-essential peripheral vascular beds are chronically vasoconstricted and essential vascular beds remain vasodilated to sustain the blood flow to vital organs. The result of brain sparing is thus region-dependent in essential and non-essential vascular beds and organs. A handful of studies investigated both cardiac and vascular outcomes in the same animals, and three studies investigated the function of two different vascular beds in the same animals. These studies are critical for the translatability of these treatments as they reveal how FGR and brain sparing regionally impact the cardiovascular system. Herrera et al. [53] showed that FGR differentially impacts how each blood vessel responds to the NO donor SNP, with the umbilical artery becoming more sensitive to this vasoactive agent in guinea pigs with FGR and the aorta remaining unchanged. Conversely, Krause et al. [60] and Graton et al. [48] investigated one essential vascular bed (carotid or coronary artery) and one non-essential vascular bed (femoral or mesenteric artery). Krause et al. [60] show that adverse structural and functional effects in the femoral artery are significantly more pronounced than in the carotid artery. This is important to consider with respect to treatments as the different vascular beds also show a differential response to antioxidant treatment, since they can act through several pathways and be impacted by receptor populations present in the vascular beds. Therefore, it is conceivable that an antioxidant treatment may have a positive benefit on one component of the cardiovascular system, but may have negative effects on another.

### 4.3. Timing of Intervention

The clinical and preclinical literature currently indicates that the programming of cardiovascular disease begins in utero. Therefore, an ideal treatment would be administered antenatally to prevent the programming of cardiovascular disease, and hence this systematic review only assessed antioxidant treatments delivered antenatally. In this review, two-thirds of studies began antenatal treatment before or on the same day that FGR was induced, which is justified in preclinical research for the validation of the hypothesis and showing proof-of-concept; however, this is a limitation for clinical translation. The clinical trial eligible for inclusion in this review commenced pentoxifylline therapy following the diagnosis of FGR, in accordance with standard clinical practice [36]. Therefore, there is an interval during which the foetus may experience hypoxia and hypoglycaemia before the initiation of therapeutic intervention and, in this study, pentoxifylline did not improve the umbilical or middle cerebral artery pulsatility before delivery. One-third of the included preclinical studies delayed the onset of treatment following the induction of FGR and of these studies, half of them demonstrated a protective or antioxidant effect on the cardiovascular system (melatonin, N-acetylcysteine and sildenafil). This demonstrates that antenatal antioxidants have potential to protect the cardiovascular system in FGR even when placental insufficiency and fetal hypoxia are established. It is also important to consider that 50% of FGR cases are not diagnosed until late gestation or after birth [91], limiting the feasibility of antenatal treatments. Whilst it would be ideal to treat all cases of FGR antenatally to prevent cardiovascular damage, an evaluation of the feasibility and efficacy of postnatal treatments for growth-restricted offspring would also be valuable to the field.

### 4.4. Study Bias and Limitations

Using the SYRCLE risk of bias tool for animal studies, we found that most publications (>90%; 36/38) were unclear in their blinding methods, with the remaining two publications stating that the allocation was not concealed from researchers (Table S3). Protocols for random housing allocation were unclear in 20 studies and not randomised in the other 18 studies. This is primarily a result of the hypoxia model, where pregnant animals were housed in a hypoxic chamber, meaning that housing depended on group allocation, making randomisation impossible. The reporting of detection bias was unclear in 36/38 publications, with only two publications stating that the outcome assessor was blinded during analysis. Using the Cochrane risk-of-bias tool for the human randomised controlled trial, we found an overall low risk of bias in the study design and conduct. Taken together, the risk of bias assessment highlighted bias in the preclinical research with minimal reporting across all domains. This is a shortcoming for preclinical animal research and limits the interpretation of the results from the animal studies included in this systematic review [92]. There is a possibility that the studies were less biased than determined by the rigid SYRCLE tool and the randomisation and allocation methodology was shortened for publication. However, it remains that improved reporting in preclinical research helps to ensure a smooth translation into clinical applications for the most positive interventions.

Given the wide range of data reporting and outcomes assessed for the cardiovascular system, for ease of interpretation, we separated the results for each publication to describe the antioxidant effects on the cardiovascular outcomes into four categories—no cardiovascular protection, mild cardiovascular protection (0 to <50% benefit), strong cardiovascular protection (>50% benefit) or antioxidant only. We acknowledge the limitations of this form of assessment. For example, if a study only examined the heart weight which was not normalised by antioxidant treatment, this would be categorised as no cardiovascular protection; however it is unknown (and unreported) whether the treatment was impactful in other cardiovascular system outcomes. We would also like to highlight that two studies in fetal sheep demonstrated that antenatal treatment with sildenafil improved cardiovascular function by restoring the blood pressure and femoral blood flow, which abolished the brain sparing response [56,57]. These studies were categorised as strong cardiovascular protection based on the criteria as they restored functional cardiovascular measures to control levels; however, these studies showed detrimental effects outside of the cardiovascular system by exacerbating birth weight deficits and inducing a greater degree of fetal hypoxia. This underscores the complexity of identifying an optimised treatment for FGR and emphasises the necessity for reporting multiple outcomes across multiple organs.

None of the included studies utilised microfluidic systems to assess cardiac and/or vascular alterations. Microfluidic systems can offer a controlled, high-resolution approach to study the complex cellular interactions and responses associated with FGR, enabling deeper insights into the mechanisms driving cardiovascular alterations and potentially guiding the development of targeted therapies. Thus, this should be considered as a future direction for this field of work.

## 5. Conclusions

This systematic review has revealed that antenatal antioxidant supplementation has potential for cardiovascular protection in FGR, particularly for improving the peripheral vascular function. Antenatal melatonin was the most frequently studied antioxidant intervention, with the majority of studies showing that melatonin mitigates both structural and functional deficits in the cardiovascular system of growth-restricted offspring, principally via actions to reduce oxidative stress and enhancing NO bioavailability. Similarly, several other antioxidants including nMitoQ, vitamin C and N-acetylcysteine were frequently studied and demonstrated strong protective effects on the cardiovascular system through mechanisms comparable to those of melatonin. However, substantial knowledge gaps remain and inconsistencies in data reporting and outcomes included across these studies prevents firm conclusions or nominating the antioxidant(s) ready for clinical application. Additional research in models of placental insufficiency where treatment is delayed following insult, together with the more widespread reporting of multiple outcomes and components of the cardiovascular system, are necessary prior to the potential translation of antioxidant therapy for cardiovascular protection in FGR.

## Figures and Tables

**Table 1:** Study characteristics.

Study	Species and Strain	FGR Model, Age Induced	FGR Comparator	Intervention, Dose, Age Given	Intervention Comparator
Asadi 2022 [36]	Human	*Pilot randomised controlled trial* Diagnosis of severe early-onset FGR determined by Delphi consensus at ~28–31 weeks of gestation	Not included	*Pentoxifylline* 400 mg per oz. tablet twice daily From diagnosis of FGR until delivery	Severe early-onset FGR + placebo
Al-Hasan 2013 [33]	Dunkin–Hartley guinea pigs	*Hypoxia (10.5% O_2_)* 0.74 GA (term = 65 d)	Normoxia (21% O_2_)	*N-acetylcysteine* ~580 mg/kg/day orally 0.80–0.95 GA	FGR + water
Aljunaidy 2018 [34]	Sprague–Dawley rats	*Hypoxia (11% O_2_)* 0.68 GA (term = 22 d)	Normoxia (21% O_2_)	*MitoQ loaded nanoparticles (nMitoQ)* 125 µM *i.v.* bolus 0.68 GA	FGR + saline
Allison 2016 [35]	Wistar rats	*Hypoxia (13% O_2_)* 0.29 GA (term = 21 d)	Normoxia	*Allopurinol* 30 mg/kg/day orally (jelly) 0.29 GA-birth	FGR
Botting 2020 [37] Study 1	Welsh mountain sheep	*Hypoxia (10% O_2_)* 0.72 GA (term = 145 d)	Normoxia	*MitoQ* 6 mg/kg/day *i.v.* bolus 0.72–0.95 GA	FGR + saline
Botting 2020 [37] Study 2	Bovans Brown chicken embryos	*Hypoxia (14% O_2_)* 0.05 GA (term = 21 d)	Normoxia (21% O_2_)	*MitoQ* 0.2 mg/kg/day via injection into chorioallantoic membrane 0.62–0.86 GA	FGR + water
Brain 2019 [38]	Welsh mountain sheep	*Hypoxia (10% O_2_)* 0.72 GA (term = 145 d)	Normoxia	*Vitamin C* 1.14 mmol/kg/day *i.v.* bolus 0.72–0.95 GA	FGR + saline
Camm 2021 [39]	Wistar rats	*Hypoxia (13% O_2_)* 0.29 GA (term = 21 d)	Normoxia	*Vitamin C* 0.5 mg/100 mL H_2_O orally 0.29–0.95 GA	FGR + water
Candia 2022 [40]	Sheep	*High-altitude pregnancy* Conception (term = 150 d)	Not included	*Melatonin* 10 mg/day orally (ethanol vehicle) 0.67 GA-birth	FGR + ethanol vehicle
Castillo-Melendez 2017 [41]	Border-Leicester cross merino sheep	*Single umbilical artery ligation (SUAL)* 0.71 GA (term = 147 d)	Sham surgery	*Melatonin* 6 mg/day *i.v.* 0.71 GA-birth	FGR + saline
Chai 2019 [42]	Wistar rats	*Hypoxia (10% O_2_) for 4 h daily* 0.68 GA (term = 22 d)	Normoxia	*Spermidine* 5 mg/kg/day *i.p.* 0.68–0.95 GA	FGR + saline
Chai 2023 [43]	Wistar rats	*Hypoxia (10% O_2_) for 4 h daily* 0.68 GA (term = 22 d)	Normoxia	*Spermidine* 5 mg/kg/day *i.p.* 0.68–0.95 GA	FGR + saline
Chatterjee 2023 [44]	Sprague–Dawley rats	*Hypoxia (11% O_2_)* 0.68 GA (term = 22 d)	Normoxia	*nMitoQ* 125 µmol/mL *i.v.* bolus 0.68 GA	FGR + saline
Finn-Sell 2018 [45]	Mice	*Endothelial nitric oxide synthase knockout (eNOS^−/−^)* Conception (term = 19.5 d)	C57/BL6J mice	*Pomegranate juice* 0.55 mg/kg/day orally 0.64–0.95 GA	FGR + water
Giussani 2012 [46]	Wistar rats	*Hypoxia (13% O_2_)* 0.29 GA (term = 21 d) *	Normoxia	*Vitamin C* 0.5 mg/100 mL H_2_O orally 0.29–0.95 GA	FGR + water
Gonzalez-Candia 2019 [47]	Sheep	*High-altitude pregnancy* Conception (term =150 d)	Not included	*Melatonin* 10 mg/day orally (ethanol vehicle) 0.67 GA-birth	FGR + ethanol vehicle
Graton 2024 [48]	Sprague–Dawley rats	*Hypoxia (11% O_2_)* 0.68 GA (term = 22 d)	Normoxia (21% O_2_)	*nMitoQ* 125 µM bolus *i.v.* 0.68 GA	FGR + saline
Hansell 2022 [49]	Wistar rats	*Hypoxia (10% O_2_)* 0.68 GA (term = 22 d)	Normoxia (21% O_2_)	*Melatonin* 5 mg/mL H_2_O orally 0.68–0.91 GA	FGR + water
Hashimoto 2012 [50]	Dunkin–Hartley guinea pigs	*Hypoxia (10.5% O_2_)* 0.75 GA (term = 65 d)	Normoxia	*N-acetylcysteine* 500–600 mg/kg/day orally 0.82–0.97 GA	FGR + water
Heras-Molina 2020 [51]	Purebred iberian pigs	*Nutrient restriction (50% daily requirement)* 0.31 GA (term = 112 d) *	Not included	*Linseed oil and Hydroxytyrosol* 4% of linseed oil and 1.5 mg hydroxytyrosol/kg/day 0.31 GA-birth	FGR
Heras-Molina 2021 [52]	Purebred iberian pigs	*Nutrient restriction (50% daily requirement)* 0.31 GA (term = 112 d)	Not included	*Linseed oil and Hydroxytyrosol* 4% of linseed oil and 1.5 mg hydroxytyrosol/kg/day 0.31 GA-birth	FGR
Herrera 2017 [53]	Pirbright White guinea pigs	*Progressive uterine artery occlusion* 0.52 GA (term = 67 d)	Sham surgery	*N-Acetylcysteine* 500 mg/kg/day orally 0.51–0.91 GA	FGR + water
Hula 2021 [55]	Sprague–Dawley rats	*Hypoxia (11% O_2_)* 0.68 GA (term = 22 d) *	Normoxia (21% O_2_)	*nMitoQ* 125 µM *i.v.* bolus 0.68 GA	FGR + saline
Hula 2023 [54]	Sprague–Dawley rats	*Hypoxia (11% O_2_)* 0.68 GA (term = 22 d)	Normoxia (21% O_2_)	*nMitoQ* 125 µM *i.v.* bolus 0.68 GA	FGR + saline
Inocencio 2019 [56]	Border-Leicester sheep	*SUAL* 0.60 GA (term = 148 d)	Sham surgery	*Sildenafil* 36 mg/day *i.v.* 0.62–0.85 GA	FGR + saline
Inocencio 2020 [57]	Border-Leiester sheep	*SUAL* 0.71 GA (term = 148 d)	Sham surgery	*Sildenafil* 36 mg/day *i.v.* 0.73–0.84 GA	FGR + saline
Itani 2016 [58]	Bovans Brown chicken embryos	*Hypoxia (14% O_2_)* 0.05 GA (term = 21 d)	Normoxia (21% O_2_)	*Melatonin* 1 mg/kg/day via injection into the air cell 0.62–0.86 GA	FGR + water
Itani 2017 [27]	Bovans Brown chicken embryos	*Hypoxia (14% O_2_)* 0.05 GA (term = 21 d)	Normoxia (21% O_2_)	*Sildenafil* 4 mg/kg/day via injection into the air cell 0.62–0.86 GA	FGR + water
Kane 2013 [59]	Wistar rats	*Hypoxia (13% O_2_)* 0.27 GA (term = 22 d)	Normoxia (21% O_2_)	*Vitamin C* 5 mg/mL H_2_O orally 0.27–0.91 GA	FGR + water
Krause 2019 [60]	Pirbright White guinea pigs	*Progressive uterine artery occlusion* 0.52 GA (term = 67 d)	Sham surgery	*N-Acetylcysteine* 500 mg/kg/day orally 0.51–0.91 GA	FGR + water
Lemley 2012 [61]	Western white-face sheep	*Nutrient restriction (60% daily requirement)* 50 d GA term unknown	Adequate nutrition (100% daily requirement)	*Melatonin* 5 mg/day orally 50–130 d GA	FGR + standard pellets
Mattern 2023 [62]	Dunkin–Hartley guinea pigs	*Spontaneous FGR* (body weight < 85 g and brain–liver ratio > 0.65) (term = 69 d)	All other foetuses (body weight > 85 g and brain–liver ratio < 0.65)	*Pyrroloquinoline Quinone* 0.18 mg/day/kg orally 0.51–0.94 GA	FGR + placebo
Navarova 2004 [63]	Wistar/DV rats	*Phenytoin injections* 0.33–0.86 GA (term = 21 d) *	pH-matched water injections during pregnancy	*Melatonin* 40 µg/mL orally 0–0.90 GA	FGR + water
Ornoy 2009 [64]	Rats	*Cohen diabetic sensitive rat model* Conception (term = 21 d)	Cohen diabetic sensitive rat model with regular diet	*Vitamins C and E* 30–40 mg/kg body weight/day orally 0–1.0 GA	FGR + high-sucrose low-copper diet
Parraguez 2022 [65]	Corriedale sheep	*Patagonian pasture* 0.20 GA (term = 149 d)	Not included	*Herbal vitamin C and E supplements* 580 mg/kg orally 0.23–0.67 GA	FGR
Poudel 2013 [66] Study 1	Mice	*eNOS^−/−^* Conception (term = 19.5 d) *	C57Bl/6J	*Resveratrol* 4 g/kg orally 0.03–0.95 GA	FGR + standard food
Poudel 2013 [66] Study 2	Mice	*Catechol-O-methyl transferase knockout (COMT^−/−^)* Conception (term = 19.5 d) *	C57Bl/6J	*Resveratrol* 4 g/kg orally 0.03–0.95 GA	FGR + standard food
Renshall 2018 [67] Study 1	Mice	*eNOS^−/−^* Conception (term = 19.5 d)	C57Bl/6J	*Melatonin* 5 µg/mL orally 0.64–0.95 GA	FGR + water
Renshall 2018 [67] Study 2	Mice	*Placental specific insulin-like growth factor 2 knockout (P0^+/−^)* Conception (term = 19.5 d)	P0^+/+^	*Melatonin* 5 µg/mL orally 0.64–0.95 GA	FGR + water
Spiroski 2021 [68]	Wister rats	*Hypoxia (13% O_2_)* 0.27 GA (term = 22 d)	Normoxia (21% O_2_)	*MitoQ* 500 µM/day orally 0.27–0.91 GA	FGR + water
Tare 2014 [26] Study 1	Sheep	*SUAL* 0.71 GA (term = 147 d)	Sham surgery	*Melatonin* 48 mg/day *i.v.* 0.75–0.80 GA	FGR + saline
Tare 2014 [26] Study 2	Sheep	*SUAL* 0.71 GA (term = 147 d)	Sham surgery	*Melatonin* 6 mg/day *i.v.* 0.71 GA-birth	FGR + saline
Vazquez-Gomez 2017 [69]	Purebred Iberian pigs	*Nutrient restriction (50% daily requirement)* 0.31 GA (term = 112 d) *	Not included	*Hydroxytyrosol* 1.5 mg/kg orally 0.31 GA-birth	FGR

GA—gestational age, d—days, *i.v.*—intravenous, *i.p.*—intraperitoneal. Details in the tables were only included if it was explicitly stated in the publication, i.e., if the % O_2_ used in normoxia was not stated, it was not included in the table. * denotes that the length of term pregnancy was not stated in publication and the value was taken from another publication from the same research group.

**Table 2:** Cardiovascular outcomes from included studies.

Study	Intervention	Age at Time of Assessment	Outcome Assessed	Effect of FGR	Effect of Intervention	Cardioprotective Potential
Asadi 2022 [36]	Pentoxifylline	Before delivery (~32 weeks)	umbilical artery pulsatility index	N/A	↔	No cardiovascular protection
			middle cerebral artery pulsatility index		↔	
Candia 2022 [40]	Melatonin	4- to 12-day-old	In vivo *cardiovascular function:*	N/A		Antioxidant only
			mean systemic BP at 7 and 8 days		↓	
			carotid blood flow at 9–11 days		↑	
			carotid vascular resistance at 7–11 days		↓	
			HR from 4–12 days		↔	
	
			Ex vivo *assessment of the middle cerebral artery in 12-day-old:*			
			vasoconstriction to potassium and U46619		↑	
			sensitivity to potassium and U46619		↔	
			vasoconstriction to 5Ht		↔	
			sensitivity to 5Ht		↓	
			vasodilation and sensitivity to MCh		↑	
			NO-independent vasodilation		↑	
			NO-dependent vasodilation		↔	
			vasodilation to SNP		↔	
			sensitivity to SNP		↑	
			vasodilation to melatonin		↑	
			sensitivity to melatonin		↔	
	
			*Histological analysis of the middle cerebral artery in 12-day-old:*			
			3-nitrotyrosine		↔	
			internal diameter, external diameter, media vascular area/vascular area, luminal area/wall area		↔	
Castillo-Melendez 2017 [41]	Melatonin	1-day-old	*Histological analysis of cerebral vasculature in white matter:*			Strong cardiovascular protection
			number of laminin-positive blood vessels	↓	↔	
			vascular endothelial growth factor	↓	↔	
			proliferating blood vessels	↓	↔	
			glucose transporter 1	↓	↑	
			proportion of caspase-3-positive blood vessels	↑	↓	
			pericyte coverage	↓	↑	
			astrocyte endfeet coverage	↓	↑	
			albumin extravasation	↑	↓	
			microbleeds	↑	↓	
Gonzalez-Candia 2019 [47]	Melatonin	3-day-old 7-day-old 12-day-old	In vivo *cardiovascular function:*	N/A		No cardiovascular protection
			mean pulmonary arterial pressure		↔	
			cardiac output		↔	
			pulmonary artery vascular resistance		↔	
Hansell 2022 [49]	Melatonin	0.91GA 4-month-old	*Fetal cardiovascular morphology:*			Strong cardiovascular protection
			heart weight and heart:body weight	↔	↔	
			aortic wall area, lumen area, total area, wall thickness	↔	↔	
			left ventricular wall area, lumen area and wall thickness	↔	↔	
			aortic wall:lumen area	↑	↓	
			*Protein analysis of fetal heart:*			
			Heat-shock protein 27, heat-shock protein 70 and 4-HNE	↔	↔	
			eNOS	↓	↑	
		
			*Cardiovascular morphology in 4-month-old:*			
			relative heart weight and relative left ventricle and septum weight	↑	↔	
			aortic wall area, lumen area, total area, wall thickness	↔	↔	
			left ventricular wall area, lumen area, total area and wall thickness	↔	↔	
			left ventricular wall:lumen area	↓	↑	
		
			Ex vivo *analysis of mesenteric artery function in 4-month-old:*			
			sensitivity to PE	↑	↓	
			vasoconstriction to PE	↔	↔	
			sensitivity to U46619	↑	↓	
			vasoconstriction to U46619	↔	↔	
		
			*Protein analysis of 4-month-old heart:*			
			Heat-shock protein 27, heat-shock protein 70 and 4-HNE	↔	↔	
			eNOS	↑	↔	
Itani 2016 [58]	Melatonin	0.90 GA	*Cardiovascular morphology:*			Strong cardiovascular protection
			heart weight	↓	↔	
			left ventricular wall volume and wall:lumen ratio	↓	↔	
			left ventricular lumen volume	↑	↔	
			right ventricular wall volume, lumen volume and wall:lumen ratio	↔	↔	
			total aorta area	↔	↔	
			aorta wall area	↔	↑	
			aorta lumen area	↔	↓	
			aorta wall:lumen area ratio	↓	↔	
		
			Ex vivo *analysis of cardiac function:*			
			left ventricular developed pressure	↓	↑	
			maximum rate of contraction and relaxation	↓	↔	
			Left-ventricular-end diastolic pressure	↑	↔	
			chronotropic sympathetic dominance	↑	↓	
			inotropic sympathetic dominance	↑	↓	
		
			Ex vivo *analysis of femoral artery function:*			
			vasodilation and sensitivity to SNP	↔	↔	
			sensitivity to ACh	↓	↑	
			contribution of NO-independent mechanisms to vasodilation	↓	↑	
			vasoconstriction to potassium	↑	↔	
			vasoconstriction to PE	↔	↔	
		
			*Protein analysis of fetal heart:*			
			3-nitrotyrosine and 4-HNE	↑	↓	
			superoxide dismutase and catalase	↓	↔	
			nitrate and nitrite concentration	↓	↑	
			glutathione peroxidase	↔	↑	
			vascular endothelial growth factor	↑	↓	
Lemley 2011 [61]	Melatonin	130 days GA	heart weight	↔	↔	Mild cardiovascular protection
		
			In vivo *cardiovascular function:*			
			HR	↓	↔	
			umbilical artery blood flow	↔	↑	
		
			*Histological analysis of cardiomyocytes:*			
			right or left ventricle thickness	↔	↔	
			left ventricle mononucleated cardiomyocyte area	↑	↓	
			right ventricle mononucleated cardiomyocyte area	↔	↔	
			right ventricle binucleated area	↑	↔	
			% cardiomyocyte binucleation in ventricles	↔	↔	
Navarova 2004 [63]	Melatonin	1-day-old	*Protein analysis of heart:*			No cardiovascular protection
			N-acetyl-ß-D-glucosaminidase	↔	↔	
			glutathione	↔	↔	
Renshall 2018 [67] Study 1 (eNOS^−/−^)	Melatonin	0.95 GA	umbilical artery diameter	↔	↔	No cardiovascular protection
		
			Ex vivo *analysis of umbilical artery function:*			
			vasoconstriction to U46619	↔	↔	
			vasodilation to SNP	↑	↔	
Renshall 2018 [67] Study 2 (P0^+/−^)	Melatonin	0.95 GA	umbilical artery diameter	↔	↔	Antioxidant only
		
			Ex vivo *analysis of umbilical artery function:*			
			vasoconstriction to U46619	↔	↔	
			vasodilation to SNP	↔	↑	
Tare 2014 [26] Study 1	Melatonin	0.76 GA	heart:body weight	↔	↔	Strong cardiovascular protection
		
			Ex vivo *analysis of cardiac function:*			
			HR	↔	↓	
			coronary blood flow	↔	↑	
			left ventricular developed pressure, max rate of contraction and relaxation	↑	↔	
			right ventricular developed pressure, max rate of contraction and relaxation	↔	↑	
			isoprenaline response	↔	↔	
			ischaemia-reperfusion-induced infarct area	↑	↓	
Tare 2014 [26] Study 2	Melatonin	1-day-old	Ex vivo *analysis of coronary artery function:*			Strong cardiovascular protection
			wall stiffness	↑	↓	
			vasoconstriction to U46619	↑	↓	
			vasodilation to SNP	↔	↔	
			sensitivity bradykinin	↓	↑	
			NO bioavailability	↓	↑	
		
			*Molecular analysis of coronary artery:*			
			eNOS and cyclooxygenase-2	↑	↓	
			cyclooxygenase-1, tropoelastin and collagen 1	↔	↔	
			collagen 2	↑	↔	
			collagen 3	↔	↓	
Aljunaidy 2018 [34]	nMitoQ	7-month-old 13-month-old	BP at 7/13-months	↔	↔	Strong cardiovascular protection
		
			*Echocardiography in 7-month-old males:*			
			HR	↔	↔	
			intraventricular septum in systole and diastole	↔	↔	
			left ventricular internal diameter in systole and diastole	↔	↔	
			left ventricular posterior wall in systole and diastole	↔	↔	
			ejection fraction, fractional shortening, cardiac output	↔	↔	
			left ventricular volume in systole and diastole	↔	↔	
			mitral valve A wave velocity	↓	↔	
			mitral valve deceleration time, E wave velocity and Tei index	↔	↔	
			mitral valve E/A index	↑	↔	
			pulmonary valve peak velocity	↓	↑	
		
			Ex vivo *analysis of mesenteric artery function in 7-month-old males:*			
			sensitivity to PE	↔	↓	
			contribution of NO to PE-induced vasoconstriction	↓	↑	
			sensitivity to MCh	↔	↔	
		
			*Echocardiography in 13-month-old males:*			
			HR	↔	↔	
			intraventricular septum in systole	↔	↔	
			intraventricular septum in diastole	↓	↔	
			left ventricular internal diameter in systole and diastole	↔	↔	
			left ventricular posterior wall in systole	↓	↔	
			left ventricular posterior wall in diastole	↔	↔	
			ejection fraction, fractional shortening, cardiac output	↔	↔	
			left ventricular volume in systole and diastole	↔	↔	
			mitral valve A wave velocity, deceleration time, E wave velocity and Tei index	↔	↔	
			mitral valve E/A index	↑	↓	
			pulmonary valve peak velocity	↔	↔	
		
			Ex vivo *analysis of mesenteric artery function in 13-month-old males:*			
			sensitivity to PE	↑	↑	
			contribution of NO to PE-induced vasoconstriction	↔	↔	
			sensitivity to MCh	↔	↑	
		
			*Echocardiography in 7-month-old females:*			
			HR	↔	↔	
			intraventricular septum in systole and diastole	↔	↔	
			left ventricular internal diameter in systole and diastole	↔	↔	
			left ventricular posterior wall in systole and diastole	↔	↔	
			ejection fraction, fractional shortening, cardiac output	↔	↔	
			left ventricular volume in systole and diastole	↔	↔	
			mitral valve A wave velocity, deceleration time, E wave velocity and Tei index	↔	↔	
			mitral valve E/A index	↑	↓	
			pulmonary valve peak velocity	↔	↔	
		
			Ex vivo *analysis of mesenteric artery function in 7-month-old females:*			
			sensitivity to PE	↔	↔	
			contribution of NO to PE-induced vasoconstriction	↔	↔	
			sensitivity to MCh	↔	↔	
		
			*Echocardiography in 13-month-old females:*			
			HR	↔	↔	
			intraventricular septum in systole and diastole	↔	↔	
			left ventricular internal diameter in systole	↑	↓	
			left ventricular internal diameter in diastole	↔	↔	
			left ventricular posterior wall in systole and diastole	↔	↔	
			ejection fraction	↓	↑	
			fractional shortening	↓	↑	
			cardiac output	↔	↔	
			left ventricular volume in systole	↑	↓	
			left ventricular volume in diastole	↔	↔	
			mitral valve A wave velocity	↓	↔	
			mitral valve deceleration time, E/A index and Tei index	↔	↔	
			mitral valve E wave velocity	↔	↑	
			pulmonary valve peak velocity	↔	↔	
		
			Ex vivo *analysis of mesenteric artery function in 13-month-old females:*			
			sensitivity to PE	↔	↔	
			contribution of NO to PE-induced vasoconstriction	↔	↔	
			sensitivity to MCh	↓	↑	
Chatterjee 2023 [44]	nMitoQ	4-month-old	*Cardiac mitochondrial respiration in males:*			No cardiovascular protection
			oxidative phosphorylation capacity for the N-, NS-, S-pathways and complex IV	↔	↔	
			oxidative phosphorylation coupling efficiency	↔	↔	
			cardiac mitochondrial content	↔	↔	
		
			*Cardiac mitochondrial respiration in females:*			
			oxidative phosphorylation capacity for the N-pathway and complex IV	↔	↔	
			oxidative phosphorylation capacity for the NS- and S-pathways	↓	↔	
			oxidative phosphorylation coupling efficiency	↔	↓	
			cardiac mitochondrial content	↔	↔	
Graton 2024 [48]	nMitoQ	4-month-old	Ex vivo *analysis of coronary artery function in females:*			Strong cardiovascular protection
			vasoconstriction to U46619	↑	↓	
			sensitivity to U46619	↑	↓	
		
			Ex vivo *analysis of coronary artery function in males:*			
			vasoconstriction to U46619	↔	↔	
			sensitivity to U46619	↑	↓	
		
			Ex vivo *analysis of mesenteric artery function in females:*			
			vasoconstriction to U46619	↑	↓	
			sensitivity to U46619	↔	↔	
		
			Ex vivo *analysis of mesenteric artery function in males:*			
			vasoconstriction to U46619	↔	↔	
			sensitivity to U46619	↑	↓	
		
			*Histological analysis of the mesenteric artery in females:*			
			eNOS	↔	↔	
			superoxide	↔	↔	
			3-nitrotyrosine	↓	↑	
			thromboxane prostanoid receptors	↔	↔	
		
			*Histological analysis of the mesenteric artery in males:*			
			eNOS	↔	↔	
			superoxide	↔	↔	
			3-nitrotyrosine	↔	↔	
			thromboxane prostanoid receptors	↑	↔	
Hula 2021 [55]	nMitoQ	4-month-old	heart weight and heart:body weight	↔	↔	Strong cardiovascular protection
		
			Ex vivo *analysis of cardiac function of males:*			
			pre-ischemic cardiac power	↔	↔	
			post-ischemic cardiac power	↓	↑	
			% cardiac recovery of baseline	↓	↑	
		
			*Protein analysis left ventricle of males: *			
			sarcoplasmic/endoplasmic reticulum Ca^2+^-ATPase	↔	↔	
			phospholamban	↔	↑	
			phosphorylated phospholamban	↔	↔	
			Ca^2+^/calmodulin kinase δ	↔	↓	
			phosphorylated Ca^2+^/calmodulin kinase δ	↔	↔	
			protein phosphatase 2Ce	↔	↑	
			protein kinase Cε	↔	↔	
			phosphorylated protein kinase Cε	↓	↔	
		
			Ex vivo *analysis of cardiac function of females:*			
			pre-ischemic cardiac power	↔	↔	
			post-ischemic cardiac power	↔	↔	
			% cardiac recovery of baseline	↓	↑	
		
			*Protein analysis left ventricle of females:*			
			sarcoplasmic/endoplasmic reticulum Ca^2+^-ATPase	↑	↔	
			phospholamban	↔	↔	
			phosphorylated phospholamban	↔	↑	
			Ca^2+^/calmodulin kinase δ	↔	↔	
			phosphorylated Ca^2+^/calmodulin kinase δ	↔	↔	
			protein phosphatase 2Ce	↔	↔	
			protein kinase Cε	↔	↔	
			phosphorylated protein kinase Cε	↔	↑	
Hula 2023 [54]	nMitoQ	4-month-old	Ex vivo *analysis of cardiac function:*	N/A		Antioxidant only
			pre-ischemic cardiac power		↔	
			% cardiac recovery of baseline		↔	
	
			*Protein analysis of left ventricle:*			
			endothelin A receptor		↑	
			endothelin B receptor (isoform A)		↔	
			endothelin B receptor (isoform C)		↔	
			protein kinase Cε		↔	
			phosphorylated protein kinase Cε		↔	
			protein kinase D		↔	
Brain 2019 [38]	Vitamin C	0.95 GA 9-month-old	fetal heart weight	↔	↔	Strong cardiovascular protection
			heart weight in 9-month-old	↔	↔	
		
			In vivo *cardiovascular function in 9-month-old:*			
			HR	↔	↔	
			basal BP	↑	↓	
			basal femoral artery blood flow	↑	↓	
			basal vascular conductance	↑	↓	
			femoral artery vasoconstriction to PE	↑	↓	
			femoral artery vasoconstriction to Ang II	↑	↓	
			femoral artery vasodilation to SNP	↑	↓	
			NO bioavailability	↔	↑	
		
			Ex vivo *analysis of femoral artery function in 9-month-old:*			
			vasodilation to MCh	↔	↔	
			sensitivity to MCh	↓	↑	
Camm 2021 [39]	Vitamin C	4-month-old	*Histological analysis of cerebral vasculature in the hippocampus:*			Strong cardiovascular protection
			% lectin-positive blood vessels in the CA1, CA2, CA3 and dentate gyrus	↓	↑	
Giussani 2012 [46]	Vitamin C	0.95 GA 4-month-old	*Fetal cardiovascular morphology:*			Strong cardiovascular protection
			absolute heart weight, relative heart weight, left ventricular area and right ventricular area	↔	↔	
			aorta wall:lumen area	↑	↓	
		
			*Protein analysis of fetal heart:*			
			Heat-shock protein 70	↑	↓	
		
			*Histological analysis of the fetal aorta:*			
			3-nitrotyrosine	↑	↓	
		
			*Cardiovascular morphology in 4-month-old:*			
			absolute heart weight, relative heart weight, left ventricular area and right ventricular area	↔	↔	
			aorta wall:lumen area	↔	↔	
		
			Ex vivo *analysis of cardiac function in 4-month-old:*			
			HR, left ventricular developed pressure and left-ventricular-end diastolic pressure	↔	↔	
			maximum rate of contraction, HR-pressure product and HR response to isoprenaline	↑	↓	
			HR response to carbachol	↓	↑	
		
			Ex vivo *analysis of femoral artery function in 4-month-old:*			
			vasodilation to SNP	↓	↔	
			vasodilation to MCh via NO-dependent mechanisms	↓	↑	
		
			*Protein analysis of 4-month-old heart:*			
			heat-shock protein 70	↔	↔	
Kane 2013 [59]	Vitamin C	4-month-old	In vivo *cardiovascular function:*			Strong cardiovascular protection
			HR and rate pressure product	↔	↔	
			mean arterial BP	↔	↓	
			systolic and diastolic arterial BP	↔	↔	
			mean femoral blood flow	↔	↔	
			HR variability in the time domain	↑	↓	
			sympathetic to parasympathetic dominance and baroreflex gain	↑	↓	
Al-Hasan 2013 [33]	N-Acetylcysteine	0.95 GA	heart:body weight	↑	↔	Strong cardiovascular protection
		
			*Protein analysis of fetal heart: *			
			malondialdehyde	↑	↓	
			cytochrome oxidase subunit 4	↔	↑	
			cytochrome oxidase activity	↑	↓	
Hashimoto 2012 [50]	N-Acetylcysteine	0.97 GA	heart weight	↑	↔	No cardiovascular protection
Herrera 2017 [53]	N-Acetylcysteine	0.91 GA	heart weight	↓	↑	Strong cardiovascular protection
		
			In vivo *Doppler ultrasound of umbilical artery:*			
			pulsatility and resistance index	↑	↓	
		
			Ex vivo *analysis of umbilical artery function:*			
			vasodilation to insulin	↓	↑	
			sensitivity to insulin	↔	↔	
			vasodilation to SNP	↔	↑	
			sensitivity to SNP	↑	↓	
		
			Ex vivo *analysis of aorta function:*			
			vasodilation to ACh	↓	↑	
			sensitivity to ACh	↔	↔	
			vasodilation to SNP	↔	↓	
			sensitivity to SNP	↔	↔	
		
			*Protein analysis of fetal aorta:*			
			3-nitrotyrosine	↑	↓	
Krause [60] 2019	N-Acetylcysteine	0.91 GA 8-month-old	In vivo *cardiovascular function:*			Strong cardiovascular protection
			morning carotid vascular resistance	↔	↔	
			diurnal carotid vascular resistance variability	↔	↔	
			morning femoral vascular resistance	↑	↓	
			diurnal femoral vascular resistance variability	↓	↑	
		
			Ex vivo *analysis of fetal carotid artery function:*			
			vasodilation to ACh	↔	↔	
			sensitivity to ACh	↔	↔	
			vasodilation to SNP	↔	↔	
			sensitivity to SNP	↔	↔	
		
			Ex vivo *analysis of fetal femoral artery function:*			
			vasodilation to ACh	↔	↔	
			sensitivity to ACh	↓	↔	
			vasodilation to SNP	↔	↓	
			sensitivity to SNP	↓	↔	
			Ex vivo *analysis of carotid artery function in 8-month-old:*			
			vasodilation to ACh	↓	↑	
			sensitivity to ACh	↔	↔	
			vasodilation to SNP	↓	↑	
			sensitivity to SNP	↔	↔	
			stretch–strain relationship	↔	↔	
			initial and final slope	↔	↔	
			elbow/transition point	↔	↔	
			Cauchy stress at the transition point	↔	↔	
		
			Ex vivo *analysis of femoral artery function in 8-month-old:*			
			vasodilation to ACh	↓	↑	
			sensitivity to ACh	↔	↔	
			vasodilation to SNP	↓	↑	
			sensitivity to SNP	↓	↑	
			stretch–strain relationship	↔	↔	
			initial and final slope	↔	↔	
			elbow/transition point	↓	↑	
			Cauchy stress at the transition point	↔	↔	
		
			*Carotid artery morphology in 8-month-old:*			
			intima, media and adventitia area	↔	↔	
			opening angle	↑	↓	
		
			*Femoral artery morphology in 8-month-old:*			
			intima area	↔	↔	
			media area	↑	↓	
			adventitia area	↓	↑	
			opening angle	↔	↔	
		
			*Histological analysis of aorta in 8-month-old:*			
			eNOS	↓	↑	
Inocencio 2019 [56]	Sildenafil	0.85 GA	Ex vivo *analysis of middle cerebral artery function:*			Strong cardiovascular protection
			maximal contraction to K^+^	↔	↔	
			overall vasodilation to SNP	↔	↑	
			maximal vasodilation to SNP	↔	↔	
			sensitivity to SNP	↔	↑	
			overall vasodilation to ACh	↔	↓	
			maximal vasodilation to ACh	↔	↓	
			sensitivity to ACh	↔	↔	
		
			Ex vivo *analysis of femoral artery function:*			
			maximal contraction to K^+^	↓	↔	
			overall vasodilation to SNP	↑	↓	
			maximal vasodilation to SNP	↔	↓	
			sensitivity to SNP	↔	↑	
			overall vasodilation to ACh	↔	↓	
			maximal vasodilation to ACh	↔	↓	
			sensitivity to ACh	↔	↔	
Inocencio 2020 [57]	Sildenafil	0.73–0.84 GA	In vivo cardiovascular function:			Strong cardiovascular protection
			heart rate	↔	↓	
			mean and systolic blood pressure	↑	↓	
			diastolic blood pressure	↔	↔	
			carotid blood flow	↓	↔	
			femoral blood flow	↓	↑	
Itani 2017 [27]	Sildenafil	0.90 GA	heart weight	↓	↔	Strong cardiovascular protection
		
		
			Ex vivo *analysis of femoral artery function:*			
			vasodilation to SNP	↔	↔	
			sensitivity to ACh	↓	↑	
			vasodilation to ACh	↓	↑	
			contribution of NO-independent mechanisms to vasodilation	↓	↑	
			vasoconstriction to potassium	↑	↔	
			vasoconstriction to PE	↔	↔	
		
			*Protein analysis of fetal heart:*			
			3-nitrotyrosine	↑	↔	
			4-HNE	↑	↓	
			superoxide dismutase and catalase	↓	↔	
			glutathione peroxidase	↔	↑	
			nitrate and nitrite concentration	↓	↑	
			phosphodiesterase type 5	↑	↓	
Botting 2020 [37] Study 1	MitoQ	0.95 GA 9-month-old	fetal heart weight	↓	↔	Strong cardiovascular protection
			fetal heart:body weight	↔	↔	
			heart weight in 9-month-old	↔	↔	
			heart:body weight in 9-month-old	↔	↓	
		
			Ex vivo *analysis of fetal femoral artery function:*			
			sensitivity to SNP	↓	↑	
		
			In vivo *cardiovascular function:*			
			mean and diastolic blood pressure	↑	↓	
			NO bioavailability	↔	↑	
		
			Ex vivo *analysis of femoral artery function in 9-month-old:*			
			vasodilation to SNP	↓	↑	
Botting 2020 [37] Study 2	MitoQ	0.90 GA	*Cardiac morphology*			Strong cardiovascular protection
			left ventricular lumen volume:wall volume ratio	↑	↓	
		
			Ex vivo *analysis of cardiac function:*			
			left ventricular developed pressure	↓	↑	
		
			Ex vivo *analysis of femoral artery function:*			
			sensitivity to ACh	↓	↑	
		
			*Cardiac mitochondrial function:*			
			respiratory control ratio	↓	↑	
		
			*Protein analysis of fetal heart:*			
			MitoP:MitoB ratio	↑	↓	
Spiroski 2021 [68]	MitoQ	0.91 GA 4-month-old	*Molecular analysis of fetal heart:*			Mild cardiovascular protection
			nuclear factor erythroid 2-like 2	↑	↓	
			glutathione peroxidase 1, catalase and superoxide dismutase	↔	↔	
			ryanodine receptor 2 and sarcoplasmic/endoplasmic reticulum Ca^2+^ transporting ATPase 2A	↑	↔	
			phospholamban and cyclic adenosine monophosphate-dependent protein kinase	↔	↔	
		
			In vivo *cardiovascular function in 4-month-old:*			
			mean, systolic and diastolic BP, HR and rate pressure product	↔	↔	
			femoral blood flow amplitude	↑	↓	
			mean BP response to PE	↑	↔	
			systolic BP response to PE	↑	↓	
			diastolic BP response to PE	↔	↔	
			femoral blood flow amplitude response to PE	↓	↑	
			reactive hyperemic response to PE in the femoral artery	↑	↓	
		
			Ex vivo *analysis of cardiac function at 4-month-old:*			
			contractility index	↑	↓	
			inotropic sympathetic dominance and left-ventricular-end diastolic pressure	↑	↔	
			left ventricular developed pressure, maximum rate of contraction and relaxation	↔	↔	
		
			*Molecular analysis of 4-month-old heart:*			
			nuclear factor erythroid 2-like 2	↑	↔	
			glutathione peroxidase 1	↑	↔	
			catalase	↑	↓	
			superoxide dismutase	↔	↔	
			ryanodine receptor 2	↑	↔	
			sarcoplasmic/endoplasmic reticulum Ca^2+^ transporting ATPase 2A and cyclic adenosine monophosphate-dependent protein kinase	↔	↔	
			phospholamban	↑	↔	
Chai 2019 [42]	Spermidine	7-day-old	heart weight	↓	↑	Strong cardiovascular protection
			heart:body weight	↑	↓	
		
			*Cardiac mitochondrial function:*			
			Pyruvate-induced state 3 and 4 mitochondrial oxygen consumption	↓	↑	
			mitochondrial respiratory control rate	↓	↑	
		
			*Cardiac mitochondrial morphology:*			
			Mito area and area of cell occupied by mitochondria	↑	↓	
		
			*Molecular analysis of myocardium:*			
			MFN2 and PGC-1⍺	↓	↑	
			FIS and DRP1	↑	↓	
		
			*Protein analysis of myocardium:*			
			superoxide dismutase	↓	↑	
			BAX/BCL2	↑	↓	
			MFN2 and PGC-1⍺	↓	↑	
			FIS1 and DRP1	↑	↓	
		
			*Histological analysis of heart:*			
			% binucleated cardiomyocytes	↑	↓	
			% mini-chromosome maintenance protein-positive cells	↓	↑	
			% apoptotic cells	↑	↓	
			cardiac fibrosis	↑	↓	
Chai 2023 [43]	Spermidine	1-day-old	heart weight	↓	↑	Strong cardiovascular protection
			heart:body weight	↑	↓	
		
			*Histological analysis of heart:*			
			% binucleated cardiomyocytes	↑	↓	
			% proliferating cardiomyocytes	↓	↑	
			% apoptotic cells	↑	↓	
			cardiac fibrosis	↑	↓	
		
			*Protein analysis of myocardium:*			
			superoxide dismutase and catalase	↓	↑	
			BAX/BCL2	↑	↓	
			MFN2, SIRT-1, PGC-1⍺, NRF-2 and TFAM	↓	↑	
			DRP1	↑	↓	
		
		
			*Cardiac mitochondrial function:*			
			pyruvate-induced state 3 and 4 mitochondrial oxygen consumption	↓	↑	
			mitochondrial respiratory control rate and adenosine triphosphate content	↓	↑	
		
			*Cardiac mitochondrial morphology:*			
			mito area and area of cell occupied by mitochondria	↑	↓	
			mitophagosome counts	↓	↑	
			mitochondrial fragmentation index	↑	↓	
Ornoy 2009 [64]	Vitamin C and E	1.0 GA	heart weight	↓	↔	No cardiovascular protection
			heart:body weight	↔	↔	
Parraguez 2022 [65]	Herbal vitamin C and E supplements	0.67 GA	heart weight	N/A	↓	Antioxidant only
Heras-Molina 2020 [51]	Linseed oil and Hydroxytyrosol	60-day-old 180-day-old	heart weight	N/A	↔	No cardiovascular protection
Heras-Molina 2021 [52]	Linseed oil and Hydroxytyrosol	0.89 GA	heart weight	N/A	↓	Antioxidant only
Poudel 2013 [66] Study 1 (eNOS^−/−^)	Resveratrol	0.90 GA	In vivo *ultrasound biomicroscopy of umbilical artery:*			No cardiovascular protection
			velocity time interval, velocity and mean gradient	↔	↔	
Poudel 2013 [66] Study 2 (COMT^−/−^)	Resveratrol	0.90 GA	In vivo *ultrasound biomicroscopy of umbilical artery:*			No cardiovascular protection
			velocity time interval, velocity and mean gradient	↔	↔	
Allison 2016 [35]	Allopurinol	4-month-old 15-month-old	Ex vivo *analysis of femoral artery function:*			Strong cardiovascular protection
			vasodilation to MCh in 4-month-old	↓	↔	
			vasodilation to MCh in 15-month-old	↓	↑	
		
			*Molecular analysis of descending aorta:*			
			short telomeres	↔	↓	
			long telomeres	↔	↑	
Mattern 2023 [62]	Pyrroloquinoline	0.94 GA	heart:body weight	↔	↔	Mild cardiovascular protection
		
			*Histological analysis of cardiomyocytes:*			
			right and left ventricle cardiomyocyte number	↓	↔	
			right and left ventricle mononucleated cardiomyocyte number	↓	↔	
			left and right ventricle proliferating cardiomyocytes	↑	↔	
			left and right ventricle apoptotic cardiomyocytes	↑	↓	
			left and right ventricle collagen deposition	↑	↓	
Finn-Sell 2018 [45]	Pomegranate juice	0.95 GA	umbilical artery diameter	↓	↔	No cardiovascular protection
		
			Ex vivo *analysis of umbilical artery function:*			
			vasoconstriction to potassium	↔	↔	
			vasoconstriction to U46619	↔	↑	
			vasoconstriction to ACh	↔	↑	
			vasodilation to SNP	↔	↑	
Vazquez-Gomez 2017 [69]	Hydroxytyrosol	25-day-old	heart weight:total viscerae weight	N/A	↔	No cardiovascular protection

GA—gestational age, BP—blood pressure, HR—heart rate, PE—phenylephrine, NO—nitric oxide, MCh—methacholine, AngII—angiotensin II, SNP—sodium nitroprusside, ACh—acetylcholine, 4-HNE—hydroxynonenal, eNOS—endothelial nitric oxide synthase.

## Data Availability

The datasets used during the current study are available from the corresponding author upon reasonable request.

## Supplementary Materials

The following supporting information can be downloaded at: https://www.mdpi.com/article/10.3390/antiox13111400/s1, Figure S1: Cardiovascular protection categorised by species and route of administration; Table S1: PRISMA Abstract Checklist; Table S2: PRISMA Checklist; Table S3: Risk of bias assessment.
