# Supplementary material for: The Use of Antioxidants for Cardiovascular Protection in Fetal Growth Restriction: A Systematic Review
Source: Antioxidants (Basel). 2024 Nov 15;13(11):1400. doi: 10.3390/antiox13111400 (PMC11591491; doi:10.3390/antiox13111400)
Supplement: Supplementary file 1 [file antioxidants-13-01400-s001.zip › Table S3.pdf]

Table S3. Risk of bias assessment

SYRCLE Risk of Bias Assessment for Animal Studies										
Study	Selection bias			Performance bias		Detection bias		Attrition bias	Reporting bias	Free from other bias?
	Random sequence generation	Groups similar at baseline	Allocation concealment	Random housing	Blinding of caregivers and/or examiners	Random outcome assessment	Blinding of outcome assessor	Incomplete outcome data assessed	Free from selective outcome reporting	
Al-Hasan 2013[33]	Unclear	Unclear	Unclear	No	Unclear	Unclear	Unclear	Unclear	Yes	Unclear
Aljunaidy 2018[34]	Unclear	Unclear	Unclear	No	Unclear	Unclear	Unclear	Unclear	Yes	Unclear
Allison 2016[35]	Unclear	Unclear	Unclear	No	Unclear	Unclear	Unclear	Yes	Yes	Unclear
Botting 2020[37]	Unclear	Unclear	Unclear	No	Unclear	Unclear	Unclear	Unclear	Yes	Unclear
Brain 2019[38]	Unclear	Unclear	Unclear	No	Unclear	Unclear	Unclear	Unclear	Yes	Unclear
Camm 2021[39]	Unclear	Unclear	Unclear	No	Unclear	Unclear	Yes	Unclear	Yes	Unclear
Candia 2022[40]	No	Unclear	Unclear	Unclear	Unclear	Unclear	Unclear	Yes	Yes	Unclear
Castillo-Melendez 2017[41]	Unclear	Unclear	Unclear	Unclear	Unclear	Unclear	Yes	Unclear	Yes	Unclear
Chai 2019[42]	Unclear	Unclear	Unclear	No	Unclear	Unclear	Unclear	Unclear	Yes	Unclear
Chai 2023[43]	Unclear	Unclear	Unclear	Unclear	Unclear	Unclear	Unclear	Unclear	Yes	Unclear
Chatterjee 2023[44]	Unclear	Unclear	Unclear	No	Unclear	Unclear	Unclear	Yes	Yes	Unclear
Finn-Sell 2018[45]	Unclear	Unclear	Unclear	Unclear	Unclear	Unclear	Unclear	Unclear	Yes	Unclear
Giussani 2012[46]	Unclear	Unclear	Unclear	No	Unclear	Unclear	Unclear	Unclear	Yes	Unclear
Gonzalez-Candia 2019[47]	No	Unclear	Unclear	Unclear	Unclear	Unclear	Unclear	Unclear	Yes	Unclear
Graton 2024[48]	Unclear	Unclear	Unclear	No	Unclear	Unclear	Unclear	Yes	Yes	Unclear
Hansell 2022[49]	Unclear	Unclear	Unclear	No	Unclear	Unclear	Unclear	Unclear	Yes	Unclear
Hashimoto 2012[50]	Unclear	Unclear	Unclear	No	Unclear	Unclear	Unclear	Unclear	Yes	Unclear
Heras-Molina 2020[51]	No	Unclear	No	Unclear	Unclear	Unclear	Unclear	Unclear	Yes	Unclear
Heras-Molina 2021[52]	No	Unclear	No	Unclear	Unclear	Unclear	Unclear	Unclear	Yes	Unclear
Herrera 2017[53]	Unclear	Unclear	Unclear	Unclear	Unclear	Unclear	Unclear	Unclear	Yes	Unclear
Hula 2021[55]	Unclear	Unclear	Unclear	No	Unclear	Unclear	Unclear	Yes	Yes	Unclear
Hula 2023[54]	No	Unclear	Unclear	No	Unclear	Unclear	Unclear	Yes	Yes	Unclear
Inocencio 2019[56]	Unclear	Unclear	Unclear	Unclear	Unclear	Unclear	Unclear	Unclear	Yes	Unclear
Inocencio 2020[57]	Unclear	Unclear	Unclear	Unclear	Unclear	Unclear	Unclear	Unclear	Yes	Unclear
Itani 2016[58]	Unclear	Unclear	Unclear	No	Unclear	Unclear	Unclear	Unclear	Yes	Unclear
Itani 2017[27]	Unclear	Unclear	Unclear	No	Unclear	Unclear	Unclear	Unclear	Yes	Unclear
Kane 2013[59]	Unclear	Unclear	Unclear	No	Unclear	Unclear	Unclear	Unclear	Yes	Unclear
Krause 2019[60]	Unclear	Unclear	Unclear	Unclear	Unclear	Unclear	Unclear	Unclear	Yes	Unclear

Lemley 2011[61]	Unclear	Unclear	Unclear	Unclear	Unclear	Unclear	Unclear	Unclear	Yes	Unclear
Mattern 2023[62]	No	Unclear	Unclear	Unclear	Unclear	Unclear	Unclear	Unclear	Yes	No
Navarova 2004[63]	Unclear	Unclear	Unclear	Unclear	Unclear	Unclear	Unclear	Unclear	Yes	Unclear
Ornoy 2009[64]	Unclear	Unclear	Unclear	Unclear	Unclear	Unclear	Unclear	Unclear	Unclear	Unclear
Parraguez 2022[65]	No	Unclear	Unclear	Unclear	No	Unclear	Unclear	Unclear	Yes	Unclear
Poudel 2013[66]	Unclear	Unclear	Unclear	Unclear	Unclear	Unclear	Unclear	Unclear	Yes	Unclear
Renshall 2018[67]	Unclear	Unclear	Yes	Unclear	No	Unclear	Unclear	Unclear	Yes	Unclear
Spiroski 2021[68]	Unclear	Unclear	Unclear	No	Unclear	Unclear	Unclear	Unclear	Unclear	Unclear
Tare 2014[26]	Unclear	Unclear	Unclear	Unclear	Unclear	Unclear	Unclear	Unclear	Yes	Unclear
Vazquez-Gomez 2017[69]	No	Unclear	No	Unclear	Unclear	Unclear	Unclear	Unclear	Yes	Unclear

Cochrane Risk of Bias for Human Studies

	Risk of bias arising from the randomization process	Risk of bias due to deviations from the intended interventions (effect of assignment to intervention)	Risk of bias due to deviations from the intended interventions (effect of adhering to intervention)	Missing outcome data	Risk of bias in measurement of outcome measure	Risk of bias in selection of reported results
Asadi 2022[36]	Low	Low	Low	Low	Medium	Low
