# Supplementary figures and images for: The Use of Antioxidants for Cardiovascular Protection in Fetal Growth Restriction: A Systematic Review
Source: Antioxidants (Basel). 2024 Nov 15;13(11):1400. doi: 10.3390/antiox13111400 (PMC11591491; doi:10.3390/antiox13111400)

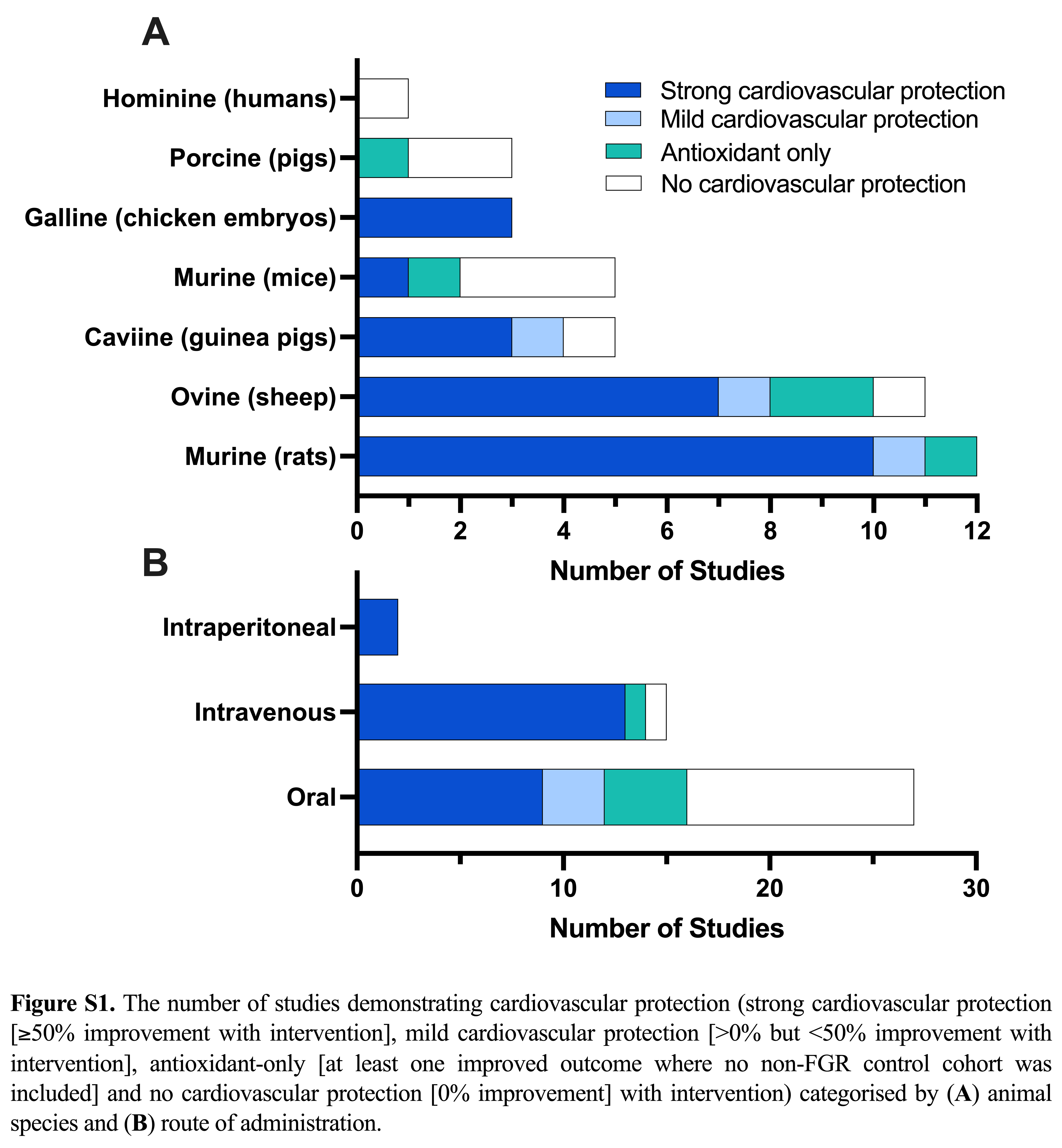

Supplement: Supplementary file 1 [file antioxidants-13-01400-s001.zip › Figure S1.tiff]
